# Combined Intake of Fish Oil and D-Fagomine Prevents High-Fat High-Sucrose Diet-Induced Prediabetes by Modulating Lipotoxicity and Protein Carbonylation in the Kidney

**DOI:** 10.3390/antiox12030751

**Published:** 2023-03-19

**Authors:** Lucía Méndez, Silvia Muñoz, Lorena Barros, Bernat Miralles-Pérez, Marta Romeu, Sara Ramos-Romero, Josep Lluís Torres, Isabel Medina

**Affiliations:** 1 Instituto de Investigaciones Marinas-Consejo Superior de Investigaciones Científicas (IIM-CSIC), Eduardo Cabello 6, E-36208 Vigo, Spain; silviam@iim.csic.es (S.M.); lorenab@iim.csic.es (L.B.); medina@iim.csic.es (I.M.); 2 Unidad de Farmacología, Facultad de Medicina y Ciencias de la Salud, Universidad Rovira i Virgili, Sant Llorenç 21, E-43201 Reus, Spain; bernat.miralles@urv.cat (B.M.-P.); marta.romeu@urv.cat (M.R.); 3 Instituto de Química Avanzada de Catalunya-Consejo Superior de Investigaciones Científicas (IQAC-CSIC), Jordi Girona 18-26, E-08034 Barcelona, Spain; sara.ramosromero@ub.edu (S.R.-R.); joseplluis.torres@iqac.csic.es (J.L.T.); 4 Departamento de Biología Celular, Fisiología e Inmunología, Facultad de Biología, Universidad de Barcelona, E-08028 Barcelona, Spain

**Keywords:** kidney protein carbonylation, kidney lipotoxicity, marine omega-3 PUFAs, D-Fagomine, high-fat and high-sucrose diet, marine natural antioxidants

## Abstract

Obesity has been recognized as a major risk factor for chronic kidney disease, insulin resistance being an early common metabolic feature in patients suffering from this syndrome. This study aims to investigate the mechanism underlying the induction of kidney dysfunction and the concomitant onset of insulin resistance by long-term high-fat and sucrose diet feeding in Sprague Dawley rats. To achieve this goal, our study analyzed renal carbonylated protein patterns, ectopic lipid accumulation and fatty acid profiles and correlated them with biometrical and biochemical measurements and other body redox status parameters. Rats fed the obesogenic diet developed a prediabetic state and incipient kidney dysfunction manifested in increased plasma urea concentration and superior levels of renal fat deposition and protein carbonylation. An obesogenic diet increased renal fat by preferentially promoting the accumulation of saturated fat, arachidonic, and docosahexaenoic fatty acids while decreasing oleic acid. Renal lipotoxicity was accompanied by selectively higher carbonylation of proteins involved in the blood pH regulation, i.e., bicarbonate reclamation and synthesis, amino acid, and glucose metabolisms, directly related to the onset of insulin resistance. This study also tested the combination of antioxidant properties of fish oil with the anti-diabetic properties of buckwheat D-Fagomine to counteract diet-induced renal alterations. Results demonstrated that bioactive compounds combined attenuated lipotoxicity, induced more favorable lipid profiles and counteracted the excessive carbonylation of proteins associated with pH regulation in the kidneys, resulting in an inhibition of the progression of the prediabetes state and kidney disease.

## 1. Introduction

Kidney disease constitutes a serious world health problem whose prevalence is rising with the spread of obesity and obesity-related disorders [1]. Among those disorders, insulin resistance (IR), together with oxidative stress and inflammation, promotes kidney disease [2]. IR is considered an early metabolic alteration in chronic kidney disease (CKD) patients and is related to increased risk for CKD in nondiabetic patients. In fact, IR emerges when the glomerular filtration rate is still within the normal range [3] and occurs at the molecular level of the kidney tissue even before the blood accumulation of nitrogenous substances, like urea and creatinine [1]. The underlying mechanism remains elusive, and the exhaustive description of these molecular changes becomes a critical issue in designing more efficient preventive/palliative treatments, besides finding accurate biomarkers for early kidney disease diagnosis.

Oxidative stress is a concomitant factor linking obesity, diabetes and CKD [4]. It is defined as a disturbance in the pro-oxidant and antioxidant balance in favor of the former, driving the disruption of redox signaling and control and/or molecular damage [5]. Among other alterations, oxidative stress can induce oxidative protein modifications, protein carbonylation being a major hallmark of oxidative stress-related disorders [6]. Protein carbonyls can be directly formed by the attack of ROS to the side chains of proline, arginine, lysine, and threonine or indirectly by adduction to the side chains of arginine, cysteine, histidine, or lysine residues of lipid peroxidation and carbohydrate oxidation products [7]. The oxidation of lipids and reducing sugars, drive the formation of a heterogeneous mixture of reactive carbonyl species (RCS), including α,β-unsaturated aldehydes, (e.g., 4-hydroxynonenal (HNE) and acrolein), keto-aldehydes (e.g., methylglyoxal and 4-oxo-nonenal), and di-aldehydes (e.g., malondialdehyde (MDA) and glyoxal). The reaction of those RCS with proteins forms advanced lipoxidation products (ALEs), which are now recognized to play relevant roles in numerous oxidative stress-related diseases [8]. Protein carbonylation often results in protein fragmentation, aggregation, and enhanced susceptibility to proteolytic digestion, leading to a loss in its functions [6]. Moreover, carbonylated proteins are also related to the inflammatory response. At least, it has been described that some reactive carbonyls-bound proteins act as damage-associated molecular patterns (DAMPs), which are endogenous danger molecules that are released from damaged or dying cells and interact with pattern recognition receptors (PRRs), activating the innate immune system [9,10,11]. Therefore, protein carbonylation is associated with various diseases, such as obesity, chronic renal failure, and diabetes, among others [7]. In a close relationship with oxidative stress and inflammation, lipotoxicity takes place. Lipotoxicity, as the ectopic accumulation of lipids in organs different from adipose tissue [12], is mainly associated with dysfunctional signaling and insulin resistance response in these non-adipose tissues. Particularly, renal ectopic lipid accumulation has been linked with kidney diseases, especially diabetic nephropathy, because lipotoxicity promotes podocyte injury, tubular damage, mesangial proliferation, endothelial activation, and the formation of macrophage-derived foam cells [13]. Some of the proposed mechanisms to explain those pathological effects are that excessive lipid accumulation alters cellular homeostasis, activates lipogenic and glycogenic cell-signaling pathways, and promotes oxidative stress, mitochondrial dysfunction, inflammation, and cell death [12,13].

It has been well established that the excessive consumption of fat, which is characteristic of the so-called “westernized” diets, is a major risk factor for CKD [14]. These high-energetic diets cause multi-tissue lipotoxicity, including an aberrant fat deposition in the kidney [15] and, thus, the previously mentioned subsequent renal alterations leading to renal injury and the development of CKD. Recent evidence indicates that the quantity and the quality of the fat accumulated in kidneys influence the severity of renal damage. In fact, lipotoxicity induced by saturated fatty acids (SFAs) causes podocyte death, while the monounsaturated (MUFAs) oleic fatty acid prevents it [16]. Moreover, rat kidneys enriched in polyunsaturated fatty acids (PUFAs), especially docosahexaenoic acid (DHA), were resistant to the progress of early-stage CKD, likely due to an improvement of the antioxidant and anti-inflammatory renal status that attenuates nephropathy [17]. Other pathological renal alterations, directly or indirectly related to lipotoxicity, induced by long-term high-energetic diet feeding are increased mitochondrial fission in tubular cells, which leads to cell apoptosis [18], and renal lysosomal dysfunction, which impairs autophagic flux and contributes to lipotoxicity [19]. High-fat diets also regulate gene expression in the kidneys. Ha et al. [14] demonstrated that a high-fat diet activates protease-activating receptor 2 (PAR2) in renal tubule epithelial cells, being an important contributor to kidney inflammation, oxidative stress, and fibrosis induced by the diet.

Increasing knowledge regarding the impact of diet on human health is resulting in a growing consumer demand for a healthy diet by means of natural, health-promoting products [20]. Numerous marine-derived nutrients and bioactive compounds have been identified as having diverse biological activities (such as an anticancer or anti-inflammatory activity), with some described to interfere with the pathogenesis of different diseases [21]. Among these marine compounds, fish oil has been reported to possess beneficial health effects, which are mainly attributed to ω-3 fatty acids, particularly to eicosapentaenoic (EPA) and docosahexaenoic (DHA) acids [22]. The EPA/DHA ratio has already been demonstrated to modulate liver carbonylome and lipid profiles, controlling liver oxidative stress and inflammation [23,24], as well as gut microbiome populations from rats fed high-fat and high-sucrose (HFHS) diets [25]. Regarding kidney disease, some authors have recently reported an improvement in kidney function and structure after EPA/DHA supplementation, less oxidative stress, inflammation, and tubulointerstitial fibrosis [26,27]. However, their effects on kidney carbonylome are highly unknown.

The combination of the bioactivities of diverse food compounds has offered very promising results in health promotion research because it allows to compensate for some individual deficiencies/adverse effects or potentiate some interesting properties of each particular bioactive [28]. For example, the combination between fish oils and proanthocyanidins from grape seed extracts showed additive and even synergistic effects in decreasing insulin resistance, modulating liver carbonylome, switching lipid and lipid mediator profiles towards less proinflammatory ones, and regulating gut microbiome populations, among others [29,30,31,32,33,34,35,36]. Another interesting bioactive compound is iminociclitol D-Fagomine (FG), a food bioactive component which can be found mainly in buckwheat-based products [37]. FG is known for its properties in improving glucose tolerance and low-grade chronic inflammation, in part because it is able to inhibit intestinal glycosidases and regulate gut microbiota populations [38,39,40]. In previous studies, the combined supplementation of FG and ω-3 PUFA altered the gut microbiome by promoting the growth of Lactobacilliales and Bifidobacteriales populations, as well as the production of short-chain fatty acids (SCFAs) in rats fed HFHS diet, while decreasing visceral adipose tissue and fasting glucose concentration, hyperinsulinemia and lobular inflammation in the liver [41].

The aim of this present study is to investigate early metabolic alterations in rat kidneys induced by the long-term feeding of a HFHS diet, which is concomitant with the onset of a prediabetic state. This study particularly addresses kidney lipotoxicity, the quantity and quality of the aberrant accumulated fat, the changes in protein carbonylation patterns, and the consequent metabolic pathways altered in the kidney. The second objective of this present study is to test if the supplementation of a HFHS diet with ω-3 EPA and DHA from fish oil, FG, or both, can exert a preventive/protective effect on lipotoxicity and protein carbonylation in kidneys. Rats fed a standard (STD) diet with fish oil and FG supplementation will be included as controls. Results will shed light on the renal metabolic alterations induced by the consumption of obesogenic diets, even before their clinical manifestation. Moreover, results can offer a deeper insight into the use of food natural antioxidants in combination with other bioactive compounds to design nutritional strategies for the prevention and palliation of kidney injury induced by diet.

## 2. Materials and Methods

### 2.1. Animals, Experimental Design and Sample Collection

A total of 72 male Sprague Dawley rats (Envigo, Indianapolis, IN, USA) aged 8–9 weeks were used in the experiment. Following the acclimatization period, the rats were randomly assigned to one of eight experimental dietary groups (nine rats each). Groups were established depending on the type of background diet they fed and the type of supplement they received. According to the background diet, half of the rats (n = 36) fed a standard diet (STD) (Teklad Global 14% Protein Rodent Maintenance Diet, Harlan Laboratories, UK), and the other half fed a high-fat high-sucrose diet (HFHS) (TD.08811 45% kcal Fat Diet, Harlan Laboratories, UK). According to the supplement received, rats can be classified into four groups: (a) control oil, which was soybean oil (STD-C and HFHS-C); (b) D-Fagomine (STD-FG and HFHS-FG); (c) fish oil (STD + ω3 and HFHS + ω3; and (d) D-Fagomine and fish oil (STD-FG + ω3 and HFHS-FG + ω3). Control and fish oils were administered by oral gavage with a gastric probe at the same dose defined in previous studies (0.8 mL/kg body weight once a week) [42]. D-Fagomine was included in the feed at a proportion previously defined as well (0.96 g/kg feed) [41]. The complete description of diets is shown in Supplementary Table S1. The fatty acid composition of the different diets is shown in Supplementary Table S2 and is the same used for research by Dasilva et al. [43].

Diets and supplements were given for 24 weeks, and rats had free access to food and water during the whole experiment. Housing conditions were: 3 rats per cage under constantly controlled conditions of temperature (22 ± 2 °C) and humidity (50 ± 10%) in a 12 h light/dark cycle. Water and feed consumption were recorded daily, and body weight was monitored weekly throughout this study.

At the end of the experiment, rats were fasted overnight, anesthetized intraperitoneally with xylazine and ketamine (10 mg/kg and 80 mg/kg body weight, respectively), and sacrificed by exsanguination. Blood was collected by cardiac puncture from each animal. Then, plasma was immediately obtained by centrifugation at 850× *g* (15 min at 4 °C) to remove erythrocytes and stored with 5 mM PMSF (protease inhibitor) at –80 °C until analysis. Both kidneys were removed, washed with 0.9% NaCl solution, weighed, and examined for macroscopic abnormalities. Then, the kidneys were snap-frozen in liquid nitrogen and stored at –80 °C until analysis. Perigonadal fat were also excised, weighed, and stored at −80 °C.

Animal experiments and all procedures rigorously adhered to the European Union guidelines for the care and management of laboratory animals. The animal study protocol was approved by the Research Council (CSIC) Subcommittee of Bioethical Issues (ref. AGL2013-49079-C2-1-R) and licensed by the regional Catalan authorities (reference no. DAAM7921).

### 2.2. Biochemical and Antioxidant System Evaluation in Blood

Plasma triglycerides, total cholesterol, and high-density lipoprotein cholesterol (HDL-C) were measured by spectrophotometric methods, as described by Bucolo et al., [44] and using SpinReact Kits (SpinReact S.A., Girona, Spain). Glucose concentration was evaluated by an Ascensia ELITE XL blood glucose meter (Bayer Consumer Care AG, Basel, Switzerland). Plasma insulin was measured using a Rat/Mouse Insulin ELISA kit (Millipore Corporation, Billerica, MA, USA) following the manufacturer’s instructions. Hemoglobin concentration was measured according to the Drabkin method [45], and the percentage of hematocrit was calculated in capillary tubes after centrifugation. Plasma urea levels were determined by using the Urea Assay Kit (Sigma, St. Louis, MO, USA) according to manufacturer’s instructions. Plasma non-enzymatic antioxidant capacity was measured as the oxygen radical absorbance capacity (ORAC) [46]. Oxidized and reduced glutathione balance (GSSG/GSH) was assayed according to Hissin and Hilf [47].

### 2.3. Renal Fat Deposition and Fatty Acid Analysis in Plasma and Kidney

Renal lipotoxicity was evaluated by quantifying the amount of fat accumulated in the kidneys and the fatty acid profiles of the fat. Briefly, total lipid amount from kidneys and plasma were extracted by a modification of Bligh and Dyer protocol [48], using dichloromethane/methanol/water (2:2:1, *v*/*v*) as extraction solvent. Total fat accumulated in kidneys was determined by gravimetric quantification and normalized by g of tissue. To obtain fatty acid profiles, 0.6 mg of the organic phase was transesterified, and total fatty acids were analyzed by gas chromatography coupled with a flame ionization detector (GC-FID, Clarus 500, PerkinElmer, Waltham, MA, USA) according to Lepage and Roy [49]. Nonadecanoic acid (19.0) was used as internal standards (IS).

FAD indexes and elongase activities from total fatty acid data of kidney and plasma were calculated as product/precursor ratio. Therefore, elongase-6 (Elovl-6) activity, enzyme that initially determines the rate of FA elongation, was calculated as Elovl-6 = [18:0/16:0]. The activities of ∆9 Stearoyl-CoA Desaturases SCD-16 and SCD-18, which regulate the desaturation of SFA to MUFA, were measured as SCD-16 = [16:1ω7/16:0], SCD-18 = [18:1ω9/18:0]. ∆4, ∆5, and ∆6 Desaturases (∆4D, ∆5D, and ∆6D), which desaturate linoleic (LA) and α-linolenic (ALA) acids to form ARA, EPA and DHA, were estimated as follows: Δ4D = [22:6ω3/22:5ω3], Δ5D = [20:4ω6/20:3ω6]; Δ6D = [20:3ω6/18:2ω6]; and Δ5D + Δ6D = [20:5ω3/18:3ω3].

### 2.4. Lipid Peroxidation Levels in Kidney

Lipid peroxidation levels in the kidney were measured through conjugated dienes hydroperoxides (intermediate lipid oxidation product) following the American Oil Chemists’ Society (AOCS) method [50]. Kidney lipids were extracted and quantified as described in 2.3. subsection. Then, conjugated dienes were measured using a spectrophotometer set at 234 nm (Beckman Coulter DU 640 Spectrophotometer UV/Vis Reader, Brea, CA, USA).

### 2.5. Extraction and Fluorescent Labeling of Renal and Plasma Protein Carbonyls

Approximately 300 mg of renal tissue was minced on ice and homogenized with a tissue homogenizer by sonication for 1 min under 0.6 s cycle and 100% of amplitude with a Labsonic M ultrasonic sonifier (Sartorius AG, Goettingen, Germany) in 25 mL of buffer (20 mM sodium phosphate, pH 6.0, 0.5 mM MgCl_2_, 1 mM EDTA) containing 10 uL/mL of ProteoBlock protease inhibitor cocktail, which comprised 100 mM AEBSF–HCl, 80 mM aprotinin, 5 mM bestatin, 1.5 mM E64, 2 mM leupeptin, and 1 mM pepstatin A. The homogenate was centrifuged at 100,000× *g* (60 min at 4 °C) to recover proteins that remain in the supernatant solution. Protein concentration was determined by bicinchoninic acid assay (BCA) [51].

To evaluate protein oxidation, the carbonyl residues generated in vivo were tagged and measured by a fluorescence-based assay, as previously described [52]. Briefly, proteins were incubated with 1 mM fluorescein-5-thiosemicarbazide (FTSC) at 37 °C for 2.5 h in the dark. Then, proteins were precipitated with 20% chilled trichloroacetic acid, centrifuged (16,000× *g*, 10 min at 20 °C), and the pellets were washed 5 times with the mixture ethanol/ethyl acetate (1:1) to remove the FTSC excess. Finally, proteins were resuspended in urea buffer (7 M urea, 2 M thiourea, 2% 3,3-cholaminopropyldimethylammonio-1-propanesulfonate (CHAPS), 0.5% Pharmalyte 3–10, 0.5% IPG 3–10 buffer, and 0.4% DTT) and stored at −80 °C until use. Protein concentration was measured by Bradford’s method [53].

### 2.6. Total and Specific Protein Carbonylation Relative Quantification

Total and individual protein carbonylation were completed as previously described [29]. Briefly, to study the global protein carbonyl levels in the kidney, an equal amount (30 μg) of FTSC-tagged protein of each sample were subjected to 12% self-made monodimensional SDS-polyacrylamide gel electrophoresis (1D SDS-PAGE) and run in a Mini-PROTEAN 3 cell (Bio-Rad, Hercules, CA, USA). To visualize the protein carbonyl levels of individual proteins, 400 μg of FTSC-labeled renal proteins were resolved in two dimensional (2D) gels. For first dimension separation according to the isoelectric point of proteins, protein samples were loaded onto 11 cm IPG 3–10 Immobiline DryStrip gels (IPG strips) by using an Ettan IPGphor II isolectric focusing system (GE Healthcare Science, Uppsala, Sweden), and protein focusing was accomplished by using the appropriate voltage/time profiles indicated in the manufacturer’s instructions (GE Healthcare, Chicago, IL, USA). After focusing, cysteines on proteins were sequentially reduced and alkylated. Then, IPG strips were run in 12% laboratory-made SDS-PAGE to separate the proteins according to their molecular weights by using an Ettan Daltsix electrophoresis system (GE Healthcare Science, Uppsala, Sweden).

FTSC-tagged proteins were visualized by exposing 1D and 2D gels to a UV transilluminator UVP BioDoc-It2 Gel Imaging System (Analytik Jena AG, Upland, CA, USA). After the fluorescent signal from FTSC bound to oxidized proteins was measured, the gels were stained overnight with Coomassie dye PhastGel Blue R-350 to visualize the total protein amount in each sample. The manipulation of gels from the same experiment in parallel and the optimization of staining and destaining cycles to minimize size changes guarantee the correct matching between the gels.

Total protein carbonylation measures were obtained by analyzing the total lane pixel intensity of 1D gels using the software LabImage 1D (Kapelan Bio-Imaging Solutions, Halle, Germany). Individual carbonylation levels of proteins were calculated by measuring the pixel intensity of the corresponding protein spot in 2D gels using the PDQuest software version 7.4 (Bio-Rad, Hercules, CA, USA). Both total and specific protein carbonylation were normalized by dividing the FTSC signal intensity in the FTSC-stained gel and the Coomassie signal intensity obtained in the corresponding Coomassie-stained gel, as previously described [29].

### 2.7. Identification of Carbonylated Proteins by NanoLC–ESI–IT–MS/MS

To identify carbonylated proteins in the kidneys, spots of interest were manually cut from the 2D gels directly onto the UV transilluminator to unequivocally assure the identification of carbonylation protein and guarantee the correct superposition of gels for quantification. After several cycles of washing with water and acetonitrile, the protein presented in each spot was submitted to tryptic digestion (0.5 µM trypsin in 50 mM NH_4_HCO_3_ buffer, pH 8, overnight at 37 °C). The consequential peptides were vacuum dried (Centrifugal Vacuum Concentrator MiniVac, GYROZEN Co., Daejeon, Republic of Korea) and dissolved in 1% formic acid.

Protein identification was accomplished by the analysis nano-LC ESI-IT-MSMS analysis of the tryptic peptides. The analysis was performed on a Dionex UltiMate 3000 Series (ThermoFisher, Rockford, IL, USA) coupled to a mass spectrometer LTQ Velos Pro with electrospray ionization (ESI) (Thermo Fisher, Rockford, IL, USA). A loading solvent of water 0.1% of formic acid with a flow rate of 10 μL/min was used to concentrate and clean injected samples on a μ-precolumn cartridge (μ-Precolumn C18 PepMap; 300 μm i.d. × 5 mm) (Thermo Scientific, San Jose, CA, USA). Peptides were separated at a flow rate of 300 nL/min along a C18 PepMap RSLC column (Acclaim PepMap RSLC C18, 2 μm, 100 Å, 75 μm i.d. × 15 cm) (Thermo Scientific, San Jose, CA, USA) set to 35 °C using a binary eluent system of water 0.1% of formic acid (phase A) and acetonitrile 0.1% formic acid (phase B). An increasing proportion of solvent B was used along a 30 min linear gradient from 5% to 40%.

Peptide MS/MS analysis was performed in positive ionization mode, with the mass spectrometer operated in data-dependent acquisition (DDA) mode. MS1 survey scan acquisition was set between 400 to 1600 Da, followed by MS/MS analysis of the 6 most intense peaks with ≥2 charge state. Fragmentation was performed in collision-induced dissociation (CID) mode with a normalized collision energy of 35% and an isolation width of 2.0 Da. A dynamic exclusion of 30 s for fragmented masses after the second fragmentation event was selected. Instrument and data acquisition were controlled through Xcalibur 2.0 and Tune 2.2 software (Thermo Fisher Scientific, Inc.).

For protein identification, raw data were searched against the *Rattus norvegicus* UniprotKB/Swiss-Prot database (downloaded on 1 October 2022) using PEAKS DB (Bioinformatics Solutions Inc., Waterloo, ON, Canada). The search criteria were stated as follows: methionine oxidation and carbamidomethylation of cysteine as fixed modifications; trypsin as proteolytic enzyme with up to 2 missed cleavage sites per peptide; and peptide precursor mass tolerance ±1.0 Da and ±0.6 Da for MS/MS fragment ions. The false discovery rate (FDR) selected for identification was maintained below 1%.

### 2.8. Gene Ontology (GO) and KEGG Pathway Enrichment Analysis of Carbonylated Proteins Identified in Kidney

GO functional enrichment analysis was conducted using the tool g:GOSt of the freely online g:Profiler (https://biit.cs.ut.ee/gprofiler (accessed on 30 December 2022)) software, by submitting gene names of the carbonylated proteins identified in the kidney and selecting *Rattus norvegicus* as organism and a significant threshold Benjamin–Hochberg FDR 0.05 [54]. KEGG pathway enrichment analysis was also performed by submitting the carbonylated protein gene list to the freely online STRING (Search Tool for the Retrieval of Interacting Genes) software version 11.5 (http://stringdb.org/ (accessed on 30 December 2022)), selecting *Rattus norvegicus*, and considering a significant enrichment when FDR < 0.05, which correspond to the *p*-value corrected for multiple testing using the Benjamini–Hochberg procedure [55].

### 2.9. Statistical Analysis

Data are mean ± standard deviation (SD). Statistical analyses were performed by two-way ANalysis Of VAriance (ANOVA) with the freely available R Studio [56] software version 1.4.1103-4. Normal distribution and homogeneity of variance were evaluated through Shapiro–Wilk’s and Levene’s test, respectively. Nonparametric Kruskal–Wallis analyses were applied when data distribution did not fit a Gaussian model or heterogeneity was found in variances. The post hoc test Tukey HD was used to compare the means and significant differences were considered when *p* < 0.05.

### 2.10. Materials and Reagents

Fish oil dietary supplement with 1:1 EPA/DHA ratio and EPA + DHA 50% of total fatty acids was obtained by mixing appropriate quantities of the commercial fish oils AFAMPES 121 EPA (AFAMSA, Vigo, Spain) and EnerZona Omega 3 RX (Milan, Italy). Soybean oil, obtained from unrefined organic soy oil after first cold pressing, was from Clearspring Ltd. (London, UK). D-Fagomine (>98%) was elaborated by Bioglane SLNE (Barcelona, Spain) and provided by Taihua Shouyue (HK) International Co. Ltd. (Hong Kong, China).

Ketamine-HCl was obtained from Merial Laboratorios S.A. (Barcelona, Spain). Xylazine (Rompun 2%) was obtained from Química Farmacéutica S.A. (Barcelona, Spain). Protease inhibitor cocktail ProteoBlock was purchased from Thermo Fisher Scientific Inc. (Rockford, IL, USA). Bicinchoninic acid (BCA) assay for protein quantification and Bio-Rad protein assay were obtained from Sigma (St. Louis, MO, USA) and Bio-Rad Laboratories (Hercules, CA, USA), respectively. Bio-Rad Laboratories also provided acrylamide and bis-N,N-methylene-bis-acrylamide. Sigma also provided the reagents dithiothreitol (DTT), iodoacetamide (IA), phenylmethylsulfonyl fluoride (PMSF), trichloroacetic acid (TCA), Tris Hydrochloride (Tris–HCl), ethylenediaminetetraacetic acid (EDTA), and CHAPS detergent. Coomassie dye PhastGel Blue R-350, Immobiline DryStrip gels (IPG strips) for isoelectric focusing (IEF) of pH range 3–10 and lengths 11, pharmalyte 3–10, IPG buffer, TEMED, and bromophenol blue were all acquired purchased from GE Healthcare Bio-Sciences AB (Uppsala, Sweden). Fluorescein-5-thiosemicarbazide (FTSC) labeling for fluorescent imaging was bought in Invitrogen (Carlsbad, CA, USA) and trypsin sequencing-grade for protein digestion in Promega (Madison, WI, USA). Internal standard of nonadecanoic acid (19:0) was purchased from Larodan Fine Chemicals (Malmö, Sweden). The rest of the reagents were of analytic/LC-MS grade.

## 3. Results and Discussion

### 3.1. Effects of the High-Fat and High-Sucrose Diet and the Supplementation with Fish Oil and D-Fagomine on Biometrical, Biochemical and General Oxidative Status

#### 3.1.1. Long-Term High-Fat and High-Sucrose Diet Feeding Induces Prediabetes and Increases Oxidative Stress and Lipotoxicity in Kidney

As shown in Table 1, HFHS-fed rats significantly showed higher values of specific rate of body mass gain, body mass index (BMI), perigonadal adipose tissue, and adiposity index, in comparison to STD-fed rats. These biometric determinations were concomitant with a statistically significant increase in plasma insulin and glucose, lower concentration of total cholesterol and HDL-Cholesterol (HDL-C), and higher percentage of fat in plasma. HFHS-diet intake provoked the ectopic accumulation of fat in erythrocytes, liver, and skeletal muscle, as previously described [43]. HFHS feeding elevated the levels of urea in plasma as well. These metabolic alterations were accompanied by worse general antioxidant status due to the HFHS intake, especially according to the GSSG/GSH ratio in erythrocytes.

**Figure 1:**
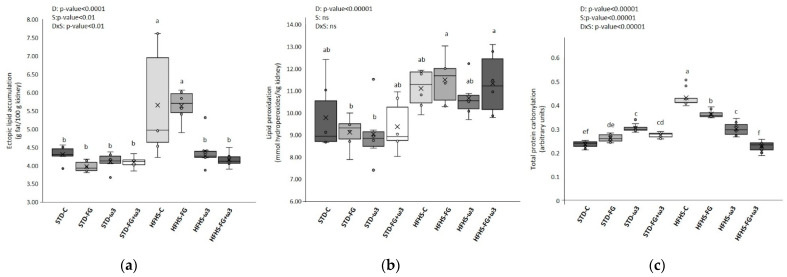
Effects of HFHS feeding and supplementation with fish oil and FG on: (**a**) ectopic lipid accumulation in kidney (g fat/100 g kidney); (**b**) lipid peroxidation levels in kidney (mmol hydroperoxides/kg kidney); (**c**) total protein carbonylation levels in kidney (arbitrary units) (data obtained from the analysis of the gels shown in Figure S1). Results were analyzed by two-way ANOVA for background diet effect (D: STD and HFHS), supplement effect (S: CONTROL, FG, ω-3, FG + ω-3), and interaction between diet and supplement (DxS), followed by Tukey HSD’s post hoc test. Data are presented as means ± SD (n = 9). Different superscripts indicate significant differences (*p* < 0.05) (analyzed by post hoc Tukey HSD). ns = no significant.

Therefore, long-term HFHS-fed rats developed a general prediabetic state, as previously described [43,57]. That state was characterized by the augment of plasma insulin concentration needed for maintaining glucose levels into the normal range, the significant increase in the perigonadal white adipose tissue and adiposity, and the general worsening of the antioxidant system. These metabolic alterations were accompanied by a significant increment of urea level in plasma in rats fed the HFHS diet, in agreement with the prediabetic state, because insulin resistance is a characteristic feature of uremia [58]. These results pointed towards a slight but relevant deterioration of renal function, which was also supported by the direct effects of the HFHS on the kidney. As Figure 1 shows, HFHS diets increased renal lipotoxicity, evaluated as the amount of ectopic fat accumulated in the kidney, and oxidative stress, demonstrated by the superior formation of lipid peroxidation products and, especially, the enhanced protein carbonylation. The stronger effect of the HFHS diet on oxidative stress parameters in the kidney, in comparison to the one found in the plasma, may be due to the presence of the anesthetics (ketamine and xylazine) in the second. Ketamine has been used in sub-anesthetic doses to create a rat model of schizophrenia because it can induce oxidative stress in brain tissues [59,60]. However, anesthetic doses of ketamine, especially in combination with xylazine, have shown some free radical scavenger activity in blood samples of sheep [61] and rabbits [62], increasing the stability of blood samples during storage. Nevertheless, some studies did not find any effects on redox blood status of sheep [63]. In this current study, the presence of the anesthetics in the plasma samples could have affected some oxidative stress measurements, and the effect of the HFHS diet could be attenuated in this fluid.

#### 3.1.2. Effect of Fish Oil and D-Fagomine on Prediabetes, Oxidative Stress, and Lipotoxicity Induced by the High-Fat and High-Sucrose Diet

As Table 1 shows, the effect of the diet, i.e., STD and HFHS, was the factor responsible for the changes in most of the biometrical and biochemical measurements of the rats, whereas the supplements had a more limited effect on them. However, rats fed FG (especially in the context of the STD diet) and its combination with fish oil in both dietary frameworks significantly reduced their specific rate of body mass gain, BMI, perigonadal adipose tissue, and adiposity index compared to the rest of the supplemented groups. These beneficial effects of the supplements were accompanied by significant decreases in the level of plasma insulin and glucose. Whole-body lipid metabolism seemed to be greater regulated by the quality of the fat’s supplement, and data reflected that fish oil, alone or in combination with FG, modulated HDL-C and plasma fat percentage. Furthermore, they tended to diminish urea in plasma when both bioactive compounds were eaten together and added to HFHS diet.

Finally, supplements were the main factor behind the changes found inoxidative stress parameters. Results revealed that the rats supplemented with fish oil, especially in combination with FG and when the HFHS was the background diet, improved their antioxidant status, showing lesser albumin carbonylation and GSSG/GSH ratios, particularly in erythrocytes and superior antioxidant capacity (ORAC values). Consequentially, the HFHS diet supplemented with the combination between fish oil and FG significantly ameliorated the whole-body oxidative stress of the rats, in agreement with the general metabolic improvement previously described for biometrical and biochemical determinations. Therefore, the combination of fish oil with FG successfully prevented the development of these alterations in HFHS-fed rats and also promoted healthier values in STD-fed ones, in comparison to corresponding controls (Table 1), supporting the protective effect of the combination of both bioactive products for developing prediabetes.

Besides the general improvement in the health status of rats, the inclusion in the HFHS diet of supplementation of fish oil, especially when it was accompanied by FG, drastically decreased renal lipotoxicity and protein carbonylation (Figure 1), likely preventing renal alterations induced by diet while ameliorating the prediabetic state.

### 3.2. Effects of the High-Fat and High-Sucrose Diet and the Supplementation with Fish Oil and D-Fagomine on Lipid Profiles

#### 3.2.1. Modulation of Lipid Profiles in Kidneys by the High-Fat and High-Sucrose Diet Intake

Results showed that long-term HFHS feeding had a strong effect on renal fatty acid composition (Table 2). This modulatory effect seemed partially due to the fatty acid composition of the HFHS diet (Table S2), particularly if it is considered the renal increase in SFAs. The fat from the HFHS diet used in this study mainly comes from milk fat, which is rich in even-numbered SFAs, such as myristic (14:0), palmitic (16:0), and stearic (18:0) [64]. It has been described that the type of fatty acid accumulated influences the severity of the metabolic alterations promoted by the ectopic fat. For instance, the lipotoxicity induced by SFAs causes insulin resistance and podocyte death [14]. Milk fat also contains odd-chain saturated fatty acids (OCFAs) [64], which can explain the rise of these fatty acids in the kidneys of HFHS-fed rats compared to the STD-fed ones.

The elevation of SFAs was also observed in the whole body, as the analysis of total fatty acid profiles in plasma demonstrated (Table 3). Results indicated that the effect of the HFHS diet was even more dramatic than in the kidney. Besides the direct influence of the fatty acid composition of the diet (Table S2), the higher increase of SFA in blood plasma, especially in the case of palmitic and stearic acids, could respond to the stimulation of de novo lipogenesis in the liver, mainly as a consequence of the high carbohydrate content of the HFHS diet [65], and, on the other hand, the decomposition of adipose tissue, which may start to overcome its lipid buffering capacity in parallel with the first stage of kidney dysfunction [66].

Regarding MUFAs, results showed significant differences between STD and HFHS-fed rats, but the direct influence of fatty acid content of the diet on these fatty acids was much lesser, at least in the kidneys (Table 2). In fact, there was a decrease in the proportion of MUFAs in HFHS-fed rats, although the background diet is richer in these fatty acids than the STD control diet. Additionally, this decrement also affected the oleic acid (18:1ω9) renal amount, despite being the main MUFA in milk fat. Some studies have demonstrated that oleic acid, which is present in other fats, typically olive oil, prevents the death of podocytes, which, in turn, can prevent and/or delay the development of kidney disease [67]. However, our results indicated that the one coming from milk was not efficiently incorporated into the kidneys, avoiding that protecting effect. On the contrary, plasma lipid profiles reproduced more faithful MUFAs diet intake, and the levels of MUFAs, especially oleic acid, were higher in HFHS-fed rats than STD-fed ones (Table 3). Interestingly, there was an increase in nervonic acid (C24:1) in plasma in the groups feeding the HFHS diet compared to STD-fed rats. Higher amounts of nervonic acid, which indicates demyelination and loss of axons, were found in patients suffering from metabolic syndrome and, at a higher concentration, in CKD patients [16].

As for PUFAs, the kidneys of STD- and HFHS-fed rats showed similar proportions of total ω6 and ω3 (Table 2). However, STD-fed rats accumulated significantly more ω6 linoleic acid (LA) than HFHS-fed ones, which can be explained by the higher content of LA in STD than HFHS chow (Table S2). On the other hand, HFHS-fed rats accumulated significantly more arachidonic acid (ARA; 20:4ω6) and dihomo-gamma-linolenic acid (DGLA; 20:3ω6), although they were not directly provided by the diet (Table S2). The modulation of ω6 PUFAs in plasma (Table 3) was different to the one described in the kidney. In plasma, the concentration of ARA and DGLA, like the total ω6 content, was higher in STD than HFHS. It has been hypothesized that this opposite behavior of the kidney to the whole body, mainly the liver and adipose tissue, can compensate for the metabolic activity of those tissues, a fact that supports the important role of the kidney in lipid metabolism control, especially when the organism is dealing with excessive levels of fatty acids [68]. With regard to ω3 PUFAs, both kidneys and plasma showed a similar modulation of lipidome in response to the diet (Table 2 and Table 3). HFHS-fed rats accumulated more EPA and DHA and showed less proportion of the precursor ALA. This higher accumulation of EPA and DHA induced by the HFHS diet was also observed in the liver and adipose tissue of the same cohort of rats [23,43]. Thus, HFHS-fed kidneys accumulated more PUFAs, especially ARA and, to a lesser extent, DHA, to the detriment of MUFAs. Considering the higher level of oxidative stress induced by the HFHS diet and the highly cytotoxic action of PUFAs lipid peroxidation products (mainly HNE from ARA, but also HHE from DHA), this enrichment in PUFAs could favor a more prooxidative environment in HFHS-kidneys, rather than offering a protective effect.

To evaluate the influence of diet on endogenous lipid metabolism, fatty acid elongase 6 (Elovl-6) activity, stearoyl-CoA (SCD), and fatty acid desaturase indexes (FAD) were measured in the kidneys and then compared with those in plasma. Results are shown in Table 4.

Elovl-6, a membrane-bound enzyme, is a condensing enzyme that converts palmitic acid (16:0) to stearic acid (18:0) and, thus, initially determines the rate of FA elongation [69]. Stearoyl-CoA desaturase (SCD), an endoplasmic reticular enzyme, biosynthesizes MUFAs from SFAs [70]. In this present study, the HFHS diet significantly increased SCD-18 in plasma, and SCD-16 and Elovl-6 followed similar tendencies. Up-regulated activities of both SCD-1 and Elovl-6, and more oleic acid levels were found in obese Zucker rats [71] and in the liver of a cyclosporine-induced nephropathy rat model [68]. In addition, the same HFHS diet increased SCD-1 indexes in adipose tissue and liver for the same cohort of rats [23,43] while increasing oleic acid too. Increased SCD-1 activities has been related to more adiposity and progression of the obesity syndrome in humans [72]. This association between SCD-1 and obesity and insulin resistance was also found in animal studies [73].

In spite of the abundant studies regarding the regulation of elongases and desaturases in fatty acid metabolism in the liver and the adipose tissue, this subject is little known in the kidney. Our results showed that the Elovl-6 activity increased significantly because of the HFHS diet in the kidney. Besides the diet, the more elongase-6 activity in the kidney may explain the increases in 18:0 and 20:0. Moreover, this higher renal elongase-6 activity seems to be addressed towards the production of long-chain SFAs rather than oleic acid, a pathway that was not activated in the kidney. As for SCD-1 activities, SCD-16 and SCD-18 indexes were significantly reduced by the HFHS diet in the kidney, in contrast to what was found in plasma. Discrepancies in lipogenic enzyme activities found in our study between the kidneys and the rest of the body have been previously described in a rat model of nephropathy [68]. It might be deduced that along with the liver and the adipose tissue, the kidney also partly contributes to lipogenic metabolism, and it may act as a compensatory lipogenic organ at the first stages of metabolic alteration. However, it is difficult to explain why SCD-1 indexes were decreased in the kidney, while increasing the Elovl-6 activity. This is because the interaction between Elovl-6 and SCD-1 in the kidney is still not clear. In the same previous study, authors also reported the opposite expression between Elovl-6 and SCD-1 in the kidney [74]. Our findings reinforce the differences in the mechanisms of regulation of the renal expression of Elovl-6 and SCD-1 and highlight the need for more investigation of them.

Additionally, the consumption of HFHS diets altered FAD indexes. There was an increment of Δ6D and a decrease of Δ5D activities in plasma, leading to the accumulation of DGLA (20:3 ω6) and lesser formation of ARA (20:4 ω6). In the kidney, HFHS diet also increased Δ6D, but Δ5D activity did not significantly diminish, explaining the superior amount of ARA in this tissue in contrast to the rest of the body. Finally, there was an up-regulation of plasma and renal Δ4D index that led to an accumulation of DHA. Additionally, superior activity of both Δ5D + Δ6D on ω3 series to produce EPA was observed in the kidneys, while it did not significantly change in plasma. FAD activities in plasma were in agreement with the ones described in the adipose tissue [43] and the liver [23]. The down-regulation of Δ5D activity found in plasma, adipose tissue and liver was the biggest difference between those tissues and the kidney regarding FAD modulation, and this discrepancy agrees with the different roles of each tissue in lipid metabolism.

#### 3.2.2. Modulation of Total Lipid Profiles in Kidneys by the Effect of Supplementation with Fish Oil and D-Fagomine

The effects of FG and fish oil supplementation on total SFAs amount were generally low, especially in the context of HFHS diets, in both the kidney (Table 2) and plasma (Table 3). In spite of this, FG supplementation increased the incorporation of OCFAs into the kidneys and elevated their levels in plasma when added to the STD diet. This may be explained by the changes induced in the gut microbiota by the FG supplementation of the STD diet, which promoted the growth of taxa that produce OCFAss, as previously reported for the same cohort of rats [41]. Increased circulating concentrations of OCFAs have been associated with lower risks of cardiometabolic diseases, and it has been reported, at least for C15:0, that they can attenuate inflammation, anemia, dyslipidemia, and fibrosis in vivo [64].

There was also a limited influence of supplements on MUFAs modulation. Only in the kidneys of STD-fed rats (Table 2), the double supplementation reduced the proportion of palmitoleic and oleic fatty acids. Several studies have reported the effect of ω3 supplementation on decreasing oleic fatty acid content in erythrocyte membranes [68], plasma, liver, and adipose tissue [31]. Moreover, rats supplemented with FG, irrespective of the background diet, decreased the concentration of nervonic acid in plasma.

The different supplements induced deep changes in renal (Table 2) and plasma (Table 3) PUFAs profiles. In general, the incorporation of fish oil into the diet, which was the main responsible for the changes, significantly decreased the amount of ω6 PUFAs and increased the ω3 in both STD and HFHS dietary contexts. Consequently, the inflammatory index ω6/ω3 was significantly reduced after fish oil supplementation. The decrease in ω6 PUFAs was mainly noticed for ARA in HFHS diets. Meanwhile, the other major ω6, LA, was scarcely altered by fish oil supplementation, with the exception of the kidneys of STD-fed rats. In these rats, LA suffered a significant drop, especially in the double supplemented group. It is worth noting that the levels of 22:4 ω6, described as a marker of the progression of CKD [16], and 22:5 ω6, significantly diminished in the kidneys of HFHS-fed rats after fish oil supplementation. As for ω3 PUFAs, there was an enrichment in DHA and, above all, EPA due to fish oil supplementation. Consequently, the EPA/DHA ratio became more balanced in the body of these rats, according to the EPA/DHA 1:1 ratio of the fish oil. Additionally, fish oil supplements significantly increased the level of docosapentaenoic acid (22:5 ω3; ω3 DPA), even if there was a negligible amount of external supplementation of this fatty acid (Table S2). A significant increment of DPA in the adipose tissue was also found after fish oil supplementation [43]. FG alone did not have any significant effect, neither on ω6 nor ω3 PUFAs in none of the tissues.

Regarding desaturases and elongases activities (Table 4), results showed that the fish oil, especially in combination with FG, significantly reduced the activities of SCD-1 when added to the STD diet. In a previous study, fish oil supplementation, alone or in combination with grape polyphenols, led to a minor activity of SCD-1 in the liver of STD-fed rats [31]. Lesser activities for SCD-1 were also found in the liver and adipose tissue for the same cohort of rats [23,43]. Rats supplemented with fish oil, with or without FG, showed significantly higher indexes of Δ5D + Δ6D and lower activity of Δ5D in tissues and dietary frameworks. Kidneys from STD-fed rats also increased Δ6D activity. Similar effects on PUFAs desaturase activities for the supplementation with fish oil, combined or not with a grape polyphenol extract, were previously described in the liver and plasma of rats fed STD and HFHS diets [31]. Those results support the consistent effect of the supplementation with ω3 from marine sources on PUFAs desaturase activity, which seems highly independent from the rest of the food composition. It is worth noting that individuals with metabolically healthy phenotype had lower estimated SCD-16 and SCD-18 activities, whereas estimated Δ6D activity was higher compared to metabolically unhealthy phenotypes [74]. The supplementation with FG alone had limited influence of FAD indexes, and there was not any significant effect.

### 3.3. Identification and Functional Enrichment Analysis of Carbonylated Proteins in the Kidney

FTSC-labeling assay and 2D gel electrophoresis (Figure S2) were used to characterize carbonylated levels of individual proteins and study in detail the possible effect of HFHS and supplements in this regard. Despite a large number of visualized protein spots on the Coomassie-stained gels (~300), only a minor portion was distinctively attached to carbonyl-specific FTSC tags showing a visible carbonylation those which are marked by numbers in the gels (Figure S2). Protein carbonylation profile was the same for all dietary groups with a total of 36 carbonylated spots visualized, analyzed, and identified by mass spectrometry, as shown in Table 5.

**Figure 2:**
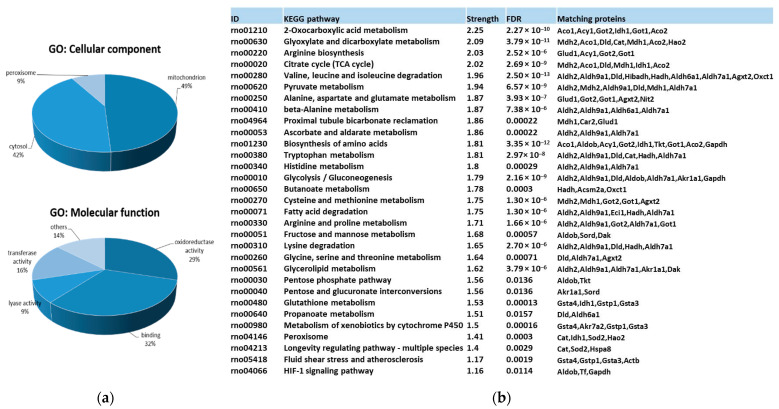
Functional enrichment analysis for carbonylated proteins identified in the kidney. (**a**) Significantly enriched GO terms according to cellular component and molecular function in carbonylated proteins of rat kidney. GO enrichment analysis were conducted by using gProfiler. (**b**) KEGG pathway enrichment analysis for carbonylated proteins in rat kidney. KEGG pathway enrichment was conducted by using STRING. Strength: Log10 (observed proteins to be annotated with a term in the interaction network created with the proteins of interest/expected proteins to be annotated in a random network of the same size). FDR: False Discovery Rate. Significant enrichment at FDR < 5%.

Gene ontology (GO) and KEGG pathway functional enrichment analysis for carbonylated proteins identified in the kidney provided a general overview of their cellular distribution, molecular function, and pathways involved. Results are summarized in Figure 2.

Protein targets for carbonylation were essentially proteins from mitochondria (48.9%), cytosol (42.2%), and peroxisome (8.9%) (Figure 2a). Carbonylated proteins located in mitochondria were phosphatidylethanolamine-binding protein 1 (Pebp1), superoxide dismutase 2 (Sod2), glutathione S-transferases (Gstp1 and Gsta4), peroxisomal trans-2-enoyl-CoA reductase (Pecr), Electron Transfer Flavoprotein Subunit Beta (Etfb), Enoyl-CoA delta isomerase 1 (Eci1), 3-Hydroxyisobutyrate dehydrogenase (Hibadh), aspartate aminotransferase 2 (Got2), malate dehydrogenase 2 (Mdh2), Hydroxyacyl-coenzyme A dehydrogenase mit. (Hadh), Sorbitol dehydrogenase (Sord), Isocitrate dehydrogenase [NADP] 1 (Idh1), Alpha-aminoadipic semialdehyde dehydrogenase (Aldh7a1), glutamate dehydrogenase 1 mit. (Glud1), Alanine--Glyoxylate Aminotransferase 2 (Agxt2), 3-Oxoacid CoA-Transferase 1 (Oxct1), Dihydrolipoamide Dehydrogenase (Dld), Methylmalonate-semialdehyde dehydrogenase [acylating] mit. (Aldh6a1), Catalase (Cat), and Aconitate hydratase 1 and 2 (Aco1 and Aco2). Cysolic proteins were: Gstp1, Triosephosphate isomerase (Tpi1), Carbonic anhydrase 2 (Ca2), omega-amidase NIT2 (Nit2), Malate dehydrogenase (Mdh1), Actin cytoplasmic 1 (Actb), Heat shock cognate 71 kDa protein (Hspa8), Fructose-bisphosphate aldolase B (Aldob), Glyceraldehyde-3-phosphate dehydrogenase (Gapdh), Alcohol dehydrogenase [NADP(+)] (Akr1a1), Sord, aspartate aminotransferase 1 (Got1), Isocitrate dehydrogenase [NADP] cytosolic (Idh1), Aldh7a1, Cat, Triokinase/FMN cyclase (Tkfc), Transketolase (Tkt), and Aco1 and 2. Finally, Quinone oxidoreductase (Cryz)/Hydroxyacid oxidase 2 (Hao2) belongs to the peroxisome. Pecr, Idh1, and Cat can be found in the peroxisome as well.

Considering the molecular function (Figure 2a), almost 92% of carbonylated proteins had catalytic activity, predominantly oxidoreductase activity (29.3%), but also transferase (16%) and lyase (9.3%) activities, followed, in less proportion, by de/hydrogenase, de/hydratase and aminocyclase activities. The 32% of carbonylated proteins showed binding capacities to diverse molecules, including nucleotides, such as NAD/NADP, toxic substances, vitamin B6, pyridoxal phosphate, ion, and sulfur. Carbonylated proteins with oxidoreductase activity in the kidney were identified as Gstp1, Pecr, Etfb, Hibadh, Mdh1, Mdh2, Gapdh, Hadh, Hao2, Akr1a1, Sord, Idh1, Aldh2, Aldh9a1, protein disulfide isomerase family A member 3 (Pdia3), Aldh7a1, Glud1, Aldh1a1, Dld, Aldh6a1, and Cat. Among those, Gapdh, Aldh2, Aldh9a1, Aldh7a1, Aldh1a1, Aldh6a1, and Dld act on the aldehyde or oxo group of donors NAD or NADP as acceptor. Proteins exhibiting transferase activity were mainly subdivided into those that transfer nitrogenous groups (Gapdh, Agxt2, and Got1 and 2) or glutathione (Gsta3, Gstp1, and Gsta4). Carbonylated proteins with lyase activity were Tpi1, Ca2, Aldob, Got1, Tkfc, and Aco1 and 2.

Finally, KEGG pathway enrichment analysis (Figure 2b) of renal carbonylated proteins indicated that carbonylation, as oxidative posttranslational modification (oxPTM), was involved in numerous signaling and metabolic processes in the kidney. Those pathways were mainly related to carbon metabolism and energy production (glycolysis/gluconeogenesis, TCA, pyruvate, and 2-oxocarboxilic acid metabolism or fatty acid degradation, among others). There was also an important enrichment in pathways devoted to amino acid metabolism and oxidative defense. Thus, carbonylation control proteins involved in glutathione metabolism and peroxisome. It is worth noting that a significant enrichment in proteins participating in the proximal tubule bicarbonate reclamation was found as well. The metabolism of xenobiotics by cytochrome P450, an important metabolic process that the kidneys are also responsible for [75], was under the control of carbonylation.

### 3.4. Quantitative Changes Induced on Renal Carbonylome by High-Fat and High-Sucrose Diet and the Effect of Fish Oil and D-Fagomine Supplementations

**Figure 3:**
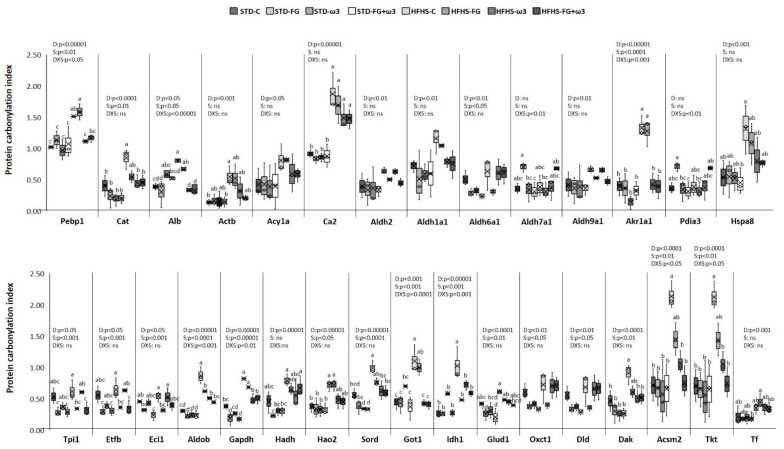
Effects of HFHS feeding and supplementation with fish oil and D-Fagomine on specific carbonylation levels of renal proteins. Results were analyzed by two-way ANOVA for background diet effect (D: STD and HFHS), supplement effect (S: CONTROL, FG, ω-3, FG + ω-3), and interaction between diet and supplement (DxS), followed by Tukey HSD’s post hoc test. Data are presented as means ± SD (n = 9). Different superscripts indicate significant differences (*p* < 0.05) (analyzed by post hoc Tukey HSD). ns = no significant.

Long-term HFHS feeding and the supplements fish oil and FG altered several proteins identified as carbonylation targets in the kidney and captured in Figure 3. The completed list of identified proteins and carbonylation indexes is detailed in Supplementary Table S3.

#### 3.4.1. Quantitative Changes Induced on Renal Carbonylome by High-Fat and High-Sucrose Diet

The intake of the HFHS diet induced significant changes in renal carbonylome according to the increased lipotoxicity found in these rats (Figure 1). In common with prior studies conducted on various tissues from animals fed HFHS diets [5,18], the increased carbonylation was highly selective for some proteins, supporting the fine metabolic control that this oxPTM can exert on cells, likely as powerful as the better-described phosphorylation or acetylation.

The strong influence of the intake of the HFHS diet on total carbonylation levels found in the kidney (Figure 1c) was reflected in the specific modulation of proteins. All the proteins responsive to HFHS diet were more carbonylated in comparison to the STD diet. Therefore, enhanced carbonylation because of the HFHS diet intake was found in twenty-four out of thirty-six protein spots (Figure 3). One of the most relevant targets was Pebp1, belonging to PEBP family of proteins, which has a high affinity towards ethanolamines and has diversified functions in maintaining cell integrity. Deregulated Pebp1 has been implicated in diabetic nephropathy through the inhibition of NF-κB activation [76]. An increased level of carbonylation of Pebp1 could partially explain that deregulation in the progression of kidney diabetic nephropathy. On the other hand, Pebp1 and Gpx4 are protein master regulators of ferroptosis. Moreover, the oxidized phosphatidyl ethanolamine 15-hydroperoxy-eicasotetraenoyl-phosphatidylethanolamine (15-HpETE-PE), coming from oxidation of ARA-PE by the activity of 15 lipoxigenase (15LO), seems to exert a triggering role in ferroptosis, and several studies have found that the PEBP1/15LO-driven ferroptosis occurs in kidney injury [77]. The higher content of ARA in kidney membranes together with the defective glutathione system indicated that HFHS kidneys could be more prone to ferroptosis than STD-fed ones. Nevertheless, the contribution of carbonylation of Pebp1 to its scaffold activity in ferroptosis during kidney injury is currently unknown [78] and needs more investigation.

Another group of proteins significantly more carbonylated in the HFHS-fed kidneys was involved in the antioxidant defense and detoxification of cytotoxic lipid peroxides. For instance, catalase was more carbonylated in HFHS-fed rats. So any diminution of activity through the oxidation of the enzyme could lead to an increase in intracellular H_2_O_2_. Catalase was among the carbonylated proteins detected in the liver of rats exhibiting higher levels of oxidative stress in a rat model of Metabolic Syndrome (SHROB) [79] and in a model induced by HFHS-diet intake [23,29,80]. HFHS diet also increased the carbonylation of proteins with ROS scavenger activity, such as albumin and actin. Increased levels of carbonylation in these proteins were previously described in the liver of rats fed HFHS [23,29,80]. Acy1a, which is involved in the degradation of N-acetylated proteins and expressed in high levels in the kidney, was more carbonylated in rats fed HFHS as well [81]. This protein has been linked to the antioxidant defense too [82]. Besides being a target of carbonylation in the kidney of Spontaneously Hypertensive rats (SHR) [83], other authors found that its expression may be redox sensitive because Acy1 was suppressed in murine models of ischaemia–reperfusion injury [84]. Other proteins significantly more carbonylated in HFHS-fed rats was the isoform 2 of the carbonic anhydrase. This isoform, the major one in the kidney, catalizes the formation of H_2_CO_3_ from CO_2_ and H_2_O for regulating blood pH. Additionally, it has been suggested that carbonic anhydrases would have an antioxidant role as well [85].

Regarding detoxification of lipid peroxides, the HFHS diet significantly enhanced carbonylation of several renal aldehyde dehydrogenase and aldo-keto reductase enzymes. Those enzymes catalyze the conversion of a variety of aldehydes, including the harmful HNE, to their corresponding acids for their detoxification [86]. Those aldehydes, which are advanced lipoxidation end-products (ALEs), are strong electrofiles, highly reactive towards nucleofile amino acids in proteins (His, Arg, and Lys) and one of the main aldehydes responsible for protein carbonylation in vivo. Their ability to impair enzyme function when they react with the protein has been well established [87]. Therefore, HFHS-induced elevation in ROS could cause losses in those aldehyde dehydrogenase functions and result in the accumulation of those aldehydes, in agreement with the above-reported lipid peroxides production in kidneys (Figure 1b). Previous studies performed in the liver of rats fed HFHS also found increased carbonylation of those aldehyde dehydogrenaes and suggested a role of these enzymes in oxidative stress-related pathologies induced by obesogenic diets [80].

The high levels of carbonylation induced by the HFHS in the kidney also affected chaperon proteins (Pdia3 and Hspa8). These enzymes are responsible for inhibiting misfolded protein aggregation and disulfide bridge formation through the reduction and isomerization of incorrect disulfide bonds to maintain the new synthesized protein native structure [88,89]. Thus, losses of their activity due to carbonylation would contribute to oxidative-induced kidney disease. They were also found as carbonylation targets in the liver of HFHS-fed rats [23,29] and in the kidney of hypertensive rats [83].

The rest of the proteins with enhanced carbonylation in HFHS-fed rat kidneys in comparison to STD-fed rats were metabolic enzymes (Figure 3), such as Tpi1, Etfb, Eci1, Aldob, Gapdh, Hadh, Hao2, Sord, Got1, Idh1, Glud1, Oxct1, Dld, Dak, Acsm2, and Tkt. Oxidative damage induced by HFHS diet on these enzymes could hamper renal energy production, as it has been previously reported in the liver of rats fed HFHS diets where the hepatic isoform of these kidney proteins was found as carbonylation targets as well [23,29]. Higher cabonylation of these kind of enzymes, as Dld, was also found in the medulla of kidneys of SHR rats [83].

#### 3.4.2. Quantitative Changes Induced on Renal Carbonylome by Fish Oil and D-Fagomine Supplementation

As Figure 3 shows, there were significant changes in carbonylome induced by the supplementation with fish oil, FG, or both. However, the magnitude of the effects of both supplements was markedly dependent on the background diet, being higher when they were added to the obesogenic diet. Interestingly, the combination of fish oil with grape polyphenols was also more effective in modulating liver carbonylated proteins in rats fed an HFHS diet than an STD one [29]. Differences in the modulation of the gut microbiota induced by each kind of diet and the influence of food matrix [25,40,41], which determine the bioavailability and bioaccessibility of the bioactive compounds, could partially explain these results. Considering the STD framework, supplements just induced significant changes in the carbonylation of four proteins compared to the STD control group. Moreover, FG had a bigger influence on renal carbonylome than fish oil on its own in this dietary group. Thus, FG, alone and in combination with fish oil, decreased carbonylation of the proteins Eci1, Gapdh, and Glud 1. The fourth protein sensitive to supplementation of STD diets was Sord, the only one responding to fish oil supplementation in STD diets. These proteins have metabolic functions, mainly participating in glucose and lipid metabolism.

Much more changes were found when supplements were added to the HFHS diet, and thirteen proteins were modulated. In contrast to what was found for the STD context, fish oil was the main supplement responsible for carbonylome changes (affecting ten proteins) rather than FG (six proteins). Moreover, the combination of both fish oil and FG was the most successful supplement to control renal carbonylome and counteract the effect of HFHS intake (affecting thirteen proteins). It is also noteworthy that all the proteins that positively responded to supplementation were proteins significantly more carbonylated due to the HFHS intake, indicating a counteracting effect of supplementation to deal with the alterations induced by the obesogenic diet in the kidney. Thus, the supplementation of the high-caloric diet with fish oil, alone, or in combination with FG, significantly decreased Pebp1 carbonylation. This decrease was accompanied by a significant reduction of ARA, which may indicate an important role of fish oils in modulating renal ferroptosis, but it needs to be further investigated. Supplements led to lesser carbonylation of proteins involved in aldehyde detoxification and ROS scavenger, and several metabolic enzymes as well, reaching carbonylation indexes similar to the ones found in the STD-group kidneys.

### 3.5. Pathways Modulated by Diet and Supplements through Carbonylome Changes in the Kidney

To fully understand the extent of the effect of HFHS intake and the antioxidant role of the supplements on renal function by modulating carbonylome, the KEGG pathway enrichment analysis for carbonylated proteins responsive to dietary interventions was conducted. Results are shown in Table 6. Due to the limited effect of supplements in modulating carbonylation in the context of STD diets, there were not any renal metabolic pathways significantly enrichement in this dietary framework. Therefore, all the pathways significantly altered by supplements and shown in Table 6 correspond to the pathways modulated in the HFHS dietary context.

#### 3.5.1. Pathways Modulated by High-Fat and High-Sucrose Diet through Carbonylome Changes in Kidney

KEGG pathway enrichment analysis of the proteins that were significantly more carbonylated because of the intake of HFHS diet, revealed that almost all the pathways identified as likely modulated by carbonylation in the kidney were affected by the diet (Table 6). Results could help understand how lipotoxicity and the subsequent oxidative stress drive kidney disease and related metabolic alterations.

One of the most relevant outcomes concerns one of the main functions of the kidney, i.e., the regulation of blood pH. To maintain systemic acid–base balance, the first step is the reclamation of all filtered bicarbonate entering the proximal tubule [90]. Interestingly, our data showed that the consumption of HFHS diets increased the carbonylation of proteins participating in this process of proximal tubule bicarbonate reclamation, likely driving to a lower capacity of buffering of kidney and, thus, the acidification of blood pH.

Results also highlighted important alterations of pathways involved in amino acid metabolism in the kidney due to the intake of HFHS diet. Renal amino acid metabolism plays a key role in the acid–base balance regulation via glutamine hydrolysis to produce “new” bicarbonate and ammonia excretion in a process known as ammoniagenesis [91]. Other amino acids different from glutamine are also used in ammoniagenesis, representing up to 20% of ammonia production in normal conditions [92]. It is likely that branched-chain amino acids (BCAA) are involved in ammoniagenesis during metabolic acidosis, and alterations of BCAA plasma profiles have been found in hyperinsulinemia [93].

The significant alteration of the amino acid metabolism induced by the HFHS diet in our study—especially the pathways related to the alanine, aspartate, and glutamate metabolism and the BCAA degradation—as well as an increase of urea and insulin in plasma, supports the deregulation that this diet induced on the renal function of blood pH regulation. Moreover, when glutamine and other amino acids are metabolized to form two ammonium ions and α-ketoglutarate, this α-ketoglutarate is metabolized to two bicarbonate anions in the TCA cycle [94]. The other resulting molecule from this cycle, malate, is an intermediate of glycolysis/gluconeogenesis, demonstrating the involvement of that pathway in the regulation of pH as well. Accordingly, both TCA and glycolysis/gluconeogenesis pathways showed significantly enhanced protein carbonylation induced by the HFHS diet.

Taken together, our results indicated that HFHS diet caused functional damage to key proteins involved in the process of blood pH regulation and the induction of metabolic acidosis, a common consequence of the intake of westernized diet. In fact, diet is the most important contributing factor to body acidity and alkalinity. Westernized diets are particularly more prone to these acid-base abnormalities and are often associated with metabolic acidosis [95]. On the other hand, acidification is considered a mechanism of insulin resistance, although the precise mechanisms are not well known. Lesser binding affinity capacity or poor recycling of insulin receptor induced by acidosis are some examples of the proposed mechanisms [92]. It is noteworthy that acidification could support a mechanism to explain the link between kidney protein carbonylation and the incipient insulin resistance detected in the HFHS-induced prediabetic rats of this current study. The increased carbonylation of the HIF-1 proteins found in the functional enrichment analysis also support it because these proteins have been involved in the insulin resistance induced by acidosis [96]. Finally, the carbonylation of proteins from the peroxisome and ascorbate and aldarate metabolism, which are involved in the antioxidant defense in kidneys, could lead to a defective renal antioxidant capacity that contributes to the general prooxidant environment.

#### 3.5.2. Pathways Modulated by Fish Oil and D-Fagomine Supplementation through Carbonylome Changes in Kidney

The inclusion of the diet of fish oil rich in EPA and DHA significantly ameliorated most of the changes in the metabolic pathways induced by the high-caloric diet (Table 6). There was a significantly decreased carbonylation in proteins involved in amino acid metabolism, especially in the alanine, aspartate, and glutamate metabolism, which was highly altered by the HFHS diet, as described above. There were also significant counteracting effects on glycolysis/gluconeogenesis, fructose and mannose metabolism, 2-oxocarboxylic acid metabolism, and the pentose phosphate pathway. Improvements in the peroxisome oxidative status and in the HIF-1 signaling pathway were also found after fish oil supplementation. This metabolic influence supports the improvements in metabolic parameters described for these rats, especially those regarding better insulin sensitivity, lipotoxicity, and oxidative stress. FG also exerted a significant effect on modulating amino acid metabolic pathways in the kidney (Table 6). In this case, FG acted mainly on essential amino acid metabolism, especially BCAA. Given the important role of BCAA on insulin resistance, the influence of FG on this pathway may be explained by the well-known antidiabetic properties of this compound. FG also had some capacity to modulate carbon metabolism, especially glycolysis/gluconeogenesis, fructose and mannose metabolism, and TCA cycle, as well as ascorbate and aldarate metabolism, peroxisome, and HIF-1 signaling. Interestingly, FG counteracted the carbonylation of proteins from the propanoate metabolism in the kidney. Although the mechanisms of SCFAs in the gut–kidney axis have not been fully investigated yet and are still controversial, they seem to exert beneficial effects on kidney function, including anti-inflammatory and anti-oxidant outcomes [97]. Previous results showed that FG promoted the growth of SCFA-producing bacteria and increased the amount of propionic acid in fecal content in the same cohort of rats in comparison with the HFHS-fed rats [25]. Therefore, the lesser carbonylation of the proteins involved in propanoate metabolism could assist the beneficial modulation exerted by propionate on renal function. The modulation of those metabolic pathways in the kidney was in agreement with the healthier status of these rats, especially the improved insulin sensitivity.

The most relevant finding was that the combination of both fish oil and FG showed additive and synergic effects on the modulation of kidney metabolic pathways through modulating carbonylation levels (Table 6). Almost all of the total individual effects were found in the combined group, but also new pathways were significantly modulated by the combination of supplements, as shown in Table 6. Those new pathways significantly enriched after the combination of both bioactive agents were arginine and proline metabolism, lysine degradation, fatty acid degradation, and butanoate metabolism. It is noteworthy that previous results indicated that the combination of fish oil and FG increased the formation of SCFAs in cecal content of the rats, including butyric and isobutyric acid [25]. The beneficial effects of butyrate on renal function have been confirmed by Felizardo et al. [98]. These authors demonstrated that butyrate preserves the glomerular basement membrane and ameliorates glomerulosclerosis and inflammation to prevent proteinuria in mice. Thus, the lesser carbonylation of this pathway found in HFHS-fed rats eating the combination of fish oil and FG may facilitate the action of these SCFAs. Therefore, HFHS-fed rats supplemented with the combination of both bioactive compounds exhibited healthier biochemical and biometrical values, the lowest lipotoxicity and whole protein carbonylation, as well as the more favorable kidney and plasma lipid profiles.

## 4. Conclusions

Long-term intake of high-fat and high-sucrose diets induced a prediabetic state in rats concomitant with significant alterations of renal function. The highly energetic diet provoked lipotoxicity in the kidneys, favoring the aberrant accumulation of SFAs and ARA but also DHA. The enrichment in PUFAs, together with the deficient antioxidant defense induced by the HFHS diet, led to more oxidative stress and the increasing formation of lipid peroxidation products and protein carbonyls. Interestingly, results reflected selective oxidative damage in renal proteins involved in the regulation of the acid–base balance. These results provide a mechanism based on the specific formation of protein carbonyls in key renal pathways that may contribute to explaining the incipient onset of insulin resistance diet-induced in rats. Additionally, the results of the present study indicated that the combination of fish oil with FG may have suppressive effects on the progression of renal dysfunction in a prediabetic state. The possible mechanisms are a) the amelioration of renal fat accumulation with the switch of lipid profiles towards less cytotoxic ones and b) the diminution of oxidative stress and oxidative damage of renal pathways mainly devoted to the regulation of blood pH and antioxidant defense. Therefore, rats fed the combination of both bioactive compounds presented a healthier phenotype with significantly improved insulin sensitivity in a mechanism closely dependent on their antioxidant properties. The information provided in this study will be useful to find new early biomarkers of kidney disease and design successfully personalized nutritional strategies for the prevention and palliation of kidney alteration induced by a diet based on the use of fish oil and FG as natural antioxidants.

## Figures and Tables

**Table 1:** General biometrical and biochemical determinations and whole-body oxidative status evaluation of Sprague Dawley rats fed the different experimental diets and supplements ^1^.

	STD-C	STD-FG	STD-ω3	STD-FG + ω3	HFHS-C	HFHS-FG	HFHS-ω3	HFHS-FG + ω3
Biometrical parameters								
Specific rate of body mass gain ^2^ (g/kg) *^$^	4.04 ^ab^ (0.76)	3.21 ^b^ (0.23)	3.97 ^ab^ (0.54)	3.34 ^b^ (0.29)	4.78 ^a^ (0.85)	4.46 ^a^ (0.40)	4.63 ^a^ (1.04)	4.33 ^a^ (0.61)
BMI ^3^ (g/cm^2^) *^$^	0.83 ^ab^ (0.07)	0.77 ^bc^ (0.03)	0.80 ^abc^ (0.04)	0.76 ^c^ (0.04)	0.85 ^ab^ (0.04)	0.83 ^ab^ (0.04)	0.83 ^a^ (0.05)	0.79 ^abc^ (0.04)
Perigonadal adipose tissue *^$^	8.99 ^bc^ (3.16)	6.51 ^c^ (1.48)	8.53 ^c^ (2.52)	7.08 ^c^ (1.89)	13.12 ^ab^ (3.92)	10.78 ^abc^ (1.36)	13.28 ^a^ (4.41)	9.91 ^abc^ (2.15)
Adiposity index (%) *^$^	1.67 ^bc^ (0.44)	1.33 ^c^ (0.28)	1.65 ^bc^ (0.38)	1.44 ^c^ (0.33)	2.37 ^a^ (0.75)	1.95 ^abc^ (0.27)	2.32 ^ab^ (0.69)	1.82 ^abc^ (0.33)
Biochemical determinations								
Plasma Insulin (ng/mL) *	0.56 ^cd^ (0.32)	0.34 ^d^ (0.08)	0.65 ^bcd^ (0.19)	0.43 ^d^ (0.20)	1.81 ^a^ (0.82)	1.36 ^abc^ (0.67)	1.46 ^abc^ (0.72)	1.41 ^abc^ (0.57)
Blood Glucose (mg/mL) *	63.00 ^b^ (4.84)	61.75 ^b^ (3.85)	63.44 ^ab^ (4.10)	63.33 ^ab^ (6.24)	70.78 ^a^ (4.99)	69.86 ^a^ (2.97)	71.33 ^a^ (5.32)	69.00 ^ab^ (3.71)
Triglycerides (mmol/L) ^ns^	0.69 (0.20)	0.61 (0.14)	0.56 (0.10)	0.53 (0.15)	0.52 (0.26)	0.57 (0.07)	0.59 (0.23)	0.42 (0.13)
Cholesterol (mmol/L) *^$#^	3.61 ^a^ (0.38)	3.30 ^a^ (0.23)	3.23 ^a^ (0.69)	3.24 ^a^ (0.55)	2.90 ^b^ (0.50)	3.15 ^ab^ (0.52)	2.55 ^b^ (0.42)	2.60 ^b^ (0.29)
HDL-C (mmol/L) *	1.15 ^a^ (0.12)	1.08 ^a^ (0.08)	1.06 ^a^ (0.18)	1.07 ^a^ (0.11)	0.94 ^b^ (0.16)	0.94 ^b^ (0.07)	0.85 ^b^ (0.12)	0.93 ^b^ (0.07)
% fat in plasma *	4.10 ^ab^ (0.87)	4.62 ^ab^ (1.08)	3.94 ^ab^ (1.54)	5.28 ^a^ (1.20)	4.00 ^ab^ (1.60)	3.43 ^ab^ (1.44)	3.31 ^ab^ (1.03)	2.87 ^b^ (1.37)
Hemoglobin (g/dL) ^ns^	17.3 (1.24)	16.86 (1.48)	16.72 (1.99)	16.32 (0.44)	16.69 (0.77)	16.76 (1.22)	16.23 (1.37)	16.19 (0.66)
Hematocrit (%) ^ns^	41.52 (3.12)	39.6 (3.26)	39.13 (4.56)	38.55 (1.33)	39.2 (1.82)	38.59 (2.63)	37.84 (3.06)	37.81 (1.85)
Plasma Urea (ng/dL) *	189.75 (11.58)	230.94 (20.71)	199.50 (94.42)	214.21 (14.72)	206.03 (40.54)	262.01 (129.97)	226.34 (37.05)	155.37 (32.00)
Oxidative status								
Albumin carbonylation index ^$#^	0.41 ^ab^ (0.04)	0.33 ^ab^ (0.05)	0.39 ^ab^ (0.11)	0.33 ^ab^ (0.05)	0.43 ^a^ (0.11)	0.42 ^ab^ (0.08)	0.32 ^b^ (0.04)	0.32 ^b^ (0.05)
Lipid peroxidation (mmol hydroperoxides/mL plasma) ^#^	0.13 (0.03)	0.14 (0.03)	0.13 (0.02)	0.16 (0.02)	0.21 (0.06)	0.17 (0.04)	0.14 (0.02)	0.14 (0.02)
ORAC (µmol TE -Trolox equivalents-/mL) ^$^	18.33 ^a^ (3.76)	17.13 ^a^ (6.82)	19.12 ^a^ (2.64)	21.49 ^b^ (1.68)	17.41 ^a^ (4.71)	18.41 ^a^ (5.73)	21.18 ^ab^ (6.12)	21.08 ^ab^ (7.62)
GSSG/GSH in plasma *^$^	3.32 ^a^ (0.48)	4.2 ^b^ (0.79)	1.98 ^c^ (0.64)	2.28 ^c^ (0.27)	2.96 ^a^ (0.85)	3.19 ^a^ (1.57)	2.72 ^a^ (0.53)	3.22 ^a^ (1.00)
GSSG/GSH in erythrocyte *^$#^	0.60 ^a^ (0.25)	0.78 ^ab^ (0.45)	1.07 ^b^ (0.34)	1.40 ^b^ (0.78)	1.48 ^b^ (1.09)	1.21 ^bc^ (0.34)	1.56 ^b^ (0.87)	0.29 ^c^ (0.11)

^1^ Two-way ANOVA analyses were conducted. * *p* < 0.05 significant differences given by the factor “diet” (D: STD and HFHS); ^$^ *p* < 0.05 significant differences given by the factor “supplement” (S: CONTROL, FG, ω-3, FG + ω-3). Superscript ^#^ indicates significant interaction (*p* < 0.05) between the factors diet and supplement (DxS). ns = no significant. Means with different superscript indicate significant differences (*p* < 0.05) (analyzed by post hoc Tukey HSD). ^2^ Specific rate of body mass gain (g/kg) = dM/Mdt, where dM represents the gain of body weight during dt = t2 − t1 and M is the rat body weight at t1. ^3^ BMI: Body Mass Index.

**Table 2:** Fatty acids composition of kidneys (mg/100 mg of total fatty acids) ^1^.

	STD-C	STD-FG	STD-ω3	STD-FG + ω3	HFHS-C	HFHS-FG	HFHS-ω3	HFHS-FG + ω3
Fatty acid								
14:0 *^$^	0.71 ^abc^ (0.28)	0.81 ^abc^ (0.19)	0.60 ^bc^ (0.05)	0.38 ^c^ (0.08)	0.83 ^ab^ (0.16)	1.05 ^a^ (0.41)	0.90 ^ab^ (0.12)	0.81 ^abc^ (0.18)
16:0 *^$#^	21.96 ^bc^ (0.21)	21.77 ^bc^ (0.70)	22.23 ^b^ (0.27)	21.21 ^c^ (0.97)	21.98 ^bc^ (0.28)	22.28 ^b^ (0.72)	23.40 ^a^ (0.43)	22.49 ^ab^ (0.47)
18:0 *^$#^	11.01 ^bc^ (3.04)	10.42 ^c^ (1.20)	12.89 ^ab^ (1.47)	14.84 ^b^ (0.66)	15.08 ^a^ (0.28)	14.99 ^a^ (0.81)	14.98 ^a^ (0.79)	15.27 ^a^ (0.54)
20:0 *^#^	0.15 ^cd^ (0.01)	0.13 ^d^ (0.01)	0.16 ^bcd^ (0.01)	0.19 ^abc^ (0.01)	0.19 ^ab^ (0.01)	0.22 ^a^ (0.03)	0.18 ^abc^ (0.00)	0.19 ^abc^ (0.02)
15:0 *^$^	0.28 ^bc^ (0.02)	0.32 ^b^ (0.04)	0.24 ^c^ (0.02)	0.30 ^bc^ (0.04)	0.42 ^a^ (0.02)	0.49 ^a^ (0.04)	0.40 ^a^ (0.03)	0.47 ^a^ (0.03)
17:0 *^$^	0.42 ^c^ (0.08)	0.51 ^bc^ (0.04)	0.44 ^c^ (0.04)	0.56 ^ab^ (0.06)	0.61 ^a^ (0.03)	0.64 ^a^ (0.04)	0.59 ^a^ (0.01)	0.65 ^a^ (0.04)
SFAs *^$#^	**34.55 ^de^ (2.74)**	**33.96 ^e^ (1.92)**	**36.7 ^cd^ (1.16)**	**37.48 ^bc^ (0.61)**	**39.16 ^ab^ (1.15)**	**39.67 ^ab^ (0.54)**	**40.54 ^a^ (0.26)**	**39.88 ^a^ (0.48)**
16:1ω7 *^$^	2.38 ^a^ (1.12)	2.12 ^a^ (0.49)	1.77 ^ab^ (0.96)	0.93 ^b^ (0.32)	1.04 ^b^ (0.24)	1.03 ^b^ (0.27)	1.00 ^b^ (0.29)	0.86 ^b^ (0.10)
18:1ω7 *^$^	3.72 ^a^ (0.57)	3.77 ^a^ (0.24)	3.29 ^ab^ (0.06)	3.05 ^bc^ (0.29)	2.93 ^bc^ (0.15)	2.97 ^c^ (0.20)	2.78 ^bc^ (0.09)	2.71 ^c^ (0.17)
18:1ω9 ^$#^	12.66 ^a^ (4.95)	12.66 ^a^ (1.66)	9.44 ^ab^ (2.94)	6.56 ^b^ (0.90)	9.83 ^ab^ (0.90)	11.66 ^a^ (2.21)	10.61 ^ab^ (1.50)	10.47 ^ab^ (1.27)
**MUFAs ^$#^**	**18.95 ^a^ (1.07)**	**18.77 ^a^ (1.03)**	**14.73 ^ab^ (2.71)**	**10.69 ^b^ (1.10)**	**14.03 ^ab^ (0.25)**	**15.93 ^ab^ (0.95)**	**14.65 ^ab^ (1.10)**	**14.28 ^ab^ (1.71)**
LA 18:2ω6 *^$#^	20.21 ^ab^ (4.75)	23.56 ^a^ (3.00)	17.30 ^bc^ (2.22)	15.74 ^c^ (2.05)	9.26 ^d^ (0.85)	9.03 ^d^ (0.99)	10.72 ^d^ (0.28)	11.09 ^d^ (0.77)
20:2ω6 *	0.37 ^a^ (0.04)	0.36 ^a^ (0.02)	0.37 ^a^ (0.06)	0.42 ^a^ (0.04)	0.16 ^b^ (0.01)	0.15 ^b^ (0.02)	0.14 ^b^ (0.01)	0.14 ^b^ (0.02)
20:3ω6 *^$^	0.61 ^c^ (0.18)	0.57 ^c^ (0.07)	0.76 ^bc^ (0.13)	0.90 ^ab^ (0.06)	0.97 ^a^ (0.12)	0.93 ^ab^ (0.08)	1.02 ^a^ (0.05)	1.07 ^a^ (0.10)
ARA 20:4ω6 *^$#^	21.46 ^cd^ (4.71)	18.83 ^d^ (3.10)	24.71 ^bcd^ (1.97)	29.23 ^ab^ (2.20)	31.67 ^a^ (1.50)	29.86 ^ab^ (3.11)	27.52 ^abc^ (0.86)	28.03 ^abc^ (1.29)
22:4ω6 *^$#^	0.85 ^a^ (0.23)	0.81 ^a^ (0.06)	0.69 ^ab^ (0.15)	0.84 ^a^ (0.15)	0.76 ^a^ (0.08)	0.77 ^a^ (0.11)	0.46 ^c^ (0.01)	0.51 ^bc^ (0.06)
22:5ω6 ^$^	0.23 ^a^ (0.01)	0.26 ^a^ (0.03)	0.21 ^ab^ (0.10)	0.16 ^ab^ (0.08)	0.27 ^a^ (0.04)	0.22 ^ab^ (0.02)	0.10 ^ab^ (0.01)	0.05 ^b^ (0.06)
ω6 *^#^	**43.80 ^abc^ (2.76)**	**44.49 ^ab^ (2.16)**	**44.1 ^ab^ (2.34)**	**47.18 ^a^ (3.19)**	**43.05 ^bc^ (1.40)**	**40.96 ^bc^ (2.27)**	**40.01 ^c^ (1.03)**	**40.83 ^bc^ (0.83)**
ALA 18:3ω3 *^$#^	0.55 ^ab^ (0.25)	0.69 ^a^ (0.14)	0.34 ^bc^ (0.03)	0.26 ^cd^ (0.09)	0.11 ^d^ (0.04)	0.15 ^d^ (0.02)	0.11 ^d^ (0.04)	0.12 ^cd^ (0.03)
18:4ω3 *^$#^	0.23 ^b^ (0.03)	0.32 ^a^ (0.05)	0.17 ^bc^ (0.09)	0.21 ^b^ (0.03)	0.13 ^c^ (0.02)	0.12 ^c^ (0.02)	0.10 ^c^ (0.00)	0.12 ^c^ (0.03)
EPA 20:5ω3 *^$#^	0.18 ^cd^ (0.16)	0.11 ^d^ (0.04)	0.44 ^b^ (0.07)	0.40 ^bc^ (0.08)	0.35 ^bc^ (0.12)	0.28 ^bcd^ (0.04)	0.93 ^a^ (0.13)	1.02 ^a^ (0.11)
DPA 22:5ω3 *^$#^	0.52 ^d^ (0.13)	0.49 ^d^ (0.03)	0.81 ^b^ (0.12)	0.95 ^a^ (0.07)	0.57 ^cd^ (0.08)	0.48 ^d^ (0.03)	0.70 ^bc^ (0.09)	0.70 ^bc^ (0.08)
DHA 22:6ω3 *^$#^	1.22 ^b^ (0.32)	1.19 ^b^ (0.17)	2.57 ^a^ (0.30)	2.71 ^a^ (0.26)	2.61 ^a^ (0.25)	2.44 ^a^ (0.23)	2.97 ^a^ (0.20)	3.04 ^a^ (0.24)
ω3 *^$^	**2.69 ^e^ (0.32)**	**2.77 ^de^ (0.13)**	**4.32 ^ab^ (0.53)**	**4.53 ^a^ (0.28)**	**3.74 ^bc^ (0.22)**	**3.43 ^ed^ (0.15)**	**4.77 ^a^ (0.41)**	**4.95 ^a^ (0.53)**
PUFAs *^$#^	**46.49 ^b^ (2.91)**	**47.26 ^b^ (1.72)**	**48.48 ^ab^ (1.08)**	**51.82 ^a^ (1.02)**	**46.79 ^b^ (1.28)**	**44.39 ^b^ (2.61)**	**44.8 ^b^ (1.73)**	**45.86 ^b^ (1.24)**
**ω6/ω3** *^$#^	16:1 ^a^ [14:1, 18:1]	16:1 ^a^ [16:1, 17:1]	10:1 ^bc^ [9:1, 12:1]	10:1 ^b^ [10:1, 13:1]	12:1 ^b^ [12:1, 12:1]	12:1 ^b^ [12:1, 12:1]	8:1 ^cd^ [8:1, 11:1]	8:1 ^d^ [8:1, 11:1]
**EPA/DHA** *^$#^	1:10 ^bc^ [1:3, 1:17]	1:12 ^bc^ [1:9, 1:15]	1:6 ^b^ [1:4, 1:7]	1:7 ^c^ [1:4, 1:10]	1:8 ^bc^ [1:5, 1:14]	1:9 ^bc^ [1:7, 1:11]	1:3 ^a^ [1:2, 1:4]	1:3 ^a^ [1:3, 1:4]
**EPA/ARA** *^$#^	1:181 ^cd^ [1:48, 1:280]	1:189 ^d^ [1:142, 1:220]	1:59 ^b^ [1:43, 1:75]	1:76 ^bc^ [1:57, 1:101]	1:100 ^bcd^ [1:56, 1:171]	1:110 ^bcd^ [1:80, 1:139]	1:30 ^a^ [1:23, 1:38]	1:28 ^a^ [1:22, 1:38]
**DHA/ARA** *^$^	1:18 ^d^ [1:15, 1:22]	1:16 ^cd^ [1:14, 1:17]	1:10 ^a^ [1:8, 1:11]	1:11 ^ab^ [1:10, 1:14]	1:12 ^bc^ [1:11, 1:15]	1:12 ^bc^ [1:12, 1:13]	1:10 ^a^ [1:9, 1:13]	1:10 ^a^ [1:9, 1:13]

^1^ Two-way ANOVA analyses were conducted, followed by Tukey HSD’s post hoc test. * *p* < 0.05 significant differences given by the factor “diet” (STD and HFHS); ^$^ *p* < 0.05 significant differences given by the factor “supplement” (CONTROL, FG, ω-3, FG + ω-3). Superscript ^#^ indicates significant interaction (*p* < 0.05) between the factors diet and supplement. Data are means (SD). Ratios are mean [minimum, maximum]. Values with different superscript indicate significant differences (*p* < 0.05) (analyzed by post hoc Tukey HSD).

**Table 3:** Total fatty acids composition of plasma (mg/100 mg of total fatty acids) ^1^.

	STD-C	STD-FG	STD-ω3	STD-FG + ω3	HFHS-C	HFHS-FG	HFHS- ω3	HFHS-FG + ω3
Fatty acid								
14:0 *	0.66 ^b^ (0.04)	0.62 ^b^ (0.06)	0.67 ^b^ (0.09)	0.63 ^b^ (0.11)	1.46 ^a^ (0.20)	1.49 ^a^ (0.13)	1.65 ^a^ (0.29)	1.51 ^a^ (0.22)
16:0 *	20.80 ^b^ (0.59)	20.45 ^b^ (0.43)	21.72 ^ab^ (1.04)	21.03 ^b^ (1.17)	23.81 ^ab^ (1.18)	23.59 ^ab^ (0.57)	22.44 ^ab^ (4.73)	24.97 ^a^ (2.72)
18:0 *	7.98 ^c^ (0.54)	8.61 ^abc^ (0.92)	8.36 ^bc^ (0.48)	8.13 ^bc^ (2.18)	9.84 ^abc^ (0.84)	10.31 ^a^ (0.78)	9.90 ^ab^ (0.73)	9.97 ^ab^ (0.78)
15:0 *	0.38 ^b^ (0.05)	0.40 ^b^ (0.03)	0.35 ^b^ (0.04)	0.37 ^b^ (0.08)	0.60 ^a^ (0.07)	0.66 ^a^ (0.05)	0.63 ^a^ (0.06)	0.69 ^a^ (0.03)
17:0 *^$#^	0.52 ^cd^ (0.07)	0.70 ^a^ (0.09)	0.48 ^d^ (0.04)	0.57 ^bcd^ (0.06)	0.60 ^abc^ (0.03)	0.63 ^ab^ (0.03)	0.61 ^abc^ (0.05)	0.65 ^ab^ (0.04)
**SFAs** *	**30.33 ^c^ (0.50)**	**30.79 ^c^ (0.97)**	**31.58 ^bc^ (0.82)**	**30.73 ^c^ (1.92)**	**36.30 ^a^ (1.48)**	**36.68 ^a^ (0.76)**	**35.23 ^ab^ (4.64)**	**37.79 ^a^ (2.38)**
16:1ω7 *	1.42 ^a^ (0.25)	1.13 ^a^ (0.12)	1.48 ^a^ (0.18)	1.40 ^a^ (0.34)	1.87 ^a^ (0.70)	1.67 ^a^ (0.38)	2.70 ^a^ (2.40)	1.37 ^a^ (0.36)
18:1ω7 *	2.55 ^a^ (0.20)	2.72 ^a^ (0.35)	2.47 ^a^ (0.16)	2.63 ^a^ (0.43)	1.85 ^b^ (0.28)	1.64 ^b^ (0.23)	1.70 ^b^ (0.33)	1.63 ^b^ (0.21)
18:1ω9 *	6.79 ^b^ (0.82)	6.23 ^b^ (0.78)	6.95 ^b^ (0.83)	6.25 ^b^ (0.51)	15.49 ^a^ (1.71)	14.95 ^a^ (0.92)	15.75 ^a^ (2.53)	14.40 ^a^ (2.29)
24:1ω9 *^$^	0.22 ^a^ (0.02)	0.19 ^a^ (0.05)	0.20 ^a^ (0.03)	0.26 ^a^ (0.10)	0.27 ^a^ (0.05)	0.20 ^a^ (0.05)	0.27 ^a^ (0.05)	0.27 ^a^ (0.03)
**MUFAs** *	**11.29 ^b^ (1.06)**	**10.47 ^b^ (1.04)**	**11.28 ^b^ (1.01)**	**10.80 ^b^ (1.07)**	**19.69 ^a^ (2.42)**	**18.69 ^a^ (1.29)**	**20.66 ^a^ (4.77)**	**17.85 ^a^ (2.25)**
LA 18:2ω6 *^$^	20.80 ^a^ (2.49)	20.62 ^a^ (1.94)	22.46 ^a^ (2.60)	21.85 ^a^ (1.56)	11.36 ^b^ (0.97)	10.66 ^b^ (0.96)	12.42 ^b^ (0.96)	12.37 ^b^ (0.42)
18:3ω6 *	0.55 ^a^ (0.08)	0.54 ^a^ (0.05)	0.41 ^abc^ (0.07)	0.49 ^ab^ (0.17)	0.35 ^bc^ (0.04)	0.36 ^bc^ (0.07)	0.33 ^c^ (0.04)	0.32 ^c^ (0.07)
20:3ω6 *^$^	0.26 ^d^ (0.04)	0.30 ^d^ (0.02)	0.39 ^cd^ (0.06)	0.38 ^cd^ (0.11)	0.61 ^ab^ (0.17)	0.52 ^bc^ (0.08)	0.73 ^a^ (0.10)	0.74 ^a^ (0.09)
ARA 20:4ω6 *^$^	32.33 ^ab^ (2.76)	33.16 ^a^ (2.46)	27.26 ^bcd^ (3.81)	29.41 ^abc^ (2.46)	26.74 ^cd^ (3.72)	27.78 ^abcd^ (1.58)	22.86 ^d^ (2.67)	23.63 ^d^ (3.64)
**ω6** *^$^	**53.95 ^ab^ (0.54)**	**54.62 ^a^ (0.76)**	**50.52 ^b^ (1.53)**	**52.13 ^ab^ (1.45)**	**39.07 ^c^ (2.85)**	**39.32 ^c^ (1.01)**	**36.34 ^c^ (1.91)**	**37.06 ^c^ (3.79)**
ALA 18:3ω3 *	0.47 ^a^ (0.11)	0.42 ^abc^ (0.06)	0.45 ^ab^ (0.08)	0.45 ^ab^ (0.12)	0.31 ^bcd^ (0.07)	0.27 ^d^ (0.03)	0.29 ^cd^ (0.07)	0.25 ^d^ (0.06)
EPA 20:5ω3 ^$#^	0.84 ^bcd^ (0.15)	0.82 ^cd^ (0.37)	1.29 ^abc^ (0.23)	1.36 ^ab^ (0.42)	0.69 ^d^ (0.14)	0.61 ^d^ (0.14)	1.74 ^a^ (0.41)	1.76 ^a^ (0.27)
DPA 22:5ω3 ^$#^	0.80 ^b^ (0.10)	0.71 ^b^ (0.11)	1.35 ^a^ (0.07)	0.90 ^b^ (0.18)	0.65 ^b^ (0.10)	1.01 ^ab^ (0.61)	0.85 ^b^ (0.11)	0.81 ^b^ (0.09)
DHA 22:6ω3 *^$^	2.32 ^c^ (0.26)	2.16 ^c^ (0.25)	3.53 ^b^ (0.49)	3.63 ^b^ (0.54)	3.29 ^b^ (0.40)	3.42 ^b^ (0.39)	4.88 ^a^ (0.49)	4.48 ^a^ (0.56)
**ω3** *^$^	**4.43 ^de^ (0.33)**	**4.12 ^e^ (0.56)**	**6.62 ^b^ (0.47)**	**6.34 ^bc^ (0.74)**	**4.94 ^de^ (0.32)**	**5.31 ^cd^ (0.55)**	**7.77 ^a^ (0.82)**	**7.30 ^ab^ (0.65)**
**PUFAs** *	**58.38 ^a^ (0.81)**	**58.74 ^a^ (0.97)**	**57.15 ^a^ (1.62)**	**58.47 ^a^ (1.20)**	**44.01 ^b^ (2.94)**	**44.64 ^b^ (1.24)**	**44.11 ^b^ (1.76)**	**44.36 ^b^ (4.25)**
**ω6/ω3** *^$#^	12:1 ^a^ [11:1, 13:1]	13:1 ^a^ [11:1, 15:1]	8:1 ^b^ [7:1, 8:1]	8:1 ^b^ [7:1, 10:1]	8:1 ^b^ [7:1, 9:1]	7:1 ^b^ [6:1, 8:1]	5:1 ^c^ [4:1, 5:1]	5:1 ^c^ [5:1, 6:1]
**EPA/DHA** *^$#^	1:3 ^ab^ [1:2, 1:3]	1:3 ^a^ [1:1, 1:5]	1:3 ^ab^ [1:2, 1:3]	1:3 ^a^ [1:2, 1:4]	1:5 ^ab^ [1:3, 1:7]	1:6 ^b^ [1:4, 1:9]	1:3 ^ab^ [1:2, 1:4]	1:3 ^a^ [1:2, 1:3]
**EPA/ARA** *^$#^	1:38 ^b^ [1:32, 1:45]	1:54 ^b^ [1:41, 1:85]	1:22 ^b^ [1:15, 1:30]	1:24 ^b^ [1:13, 1:34]	1:41 ^b^ [1:25, 1:61]	1:48 ^b^ [1:35, 1:73]	1:14 ^a^ [1:9, 1:21]	1:14 ^a^ [1:10, 1:17]
**DHA/ARA** *^$^	1:14 ^c^ [1:13, 1:15]	1:15 ^c^ [1:15, 1:18]	1:8 ^b^ [1:6, 1:9]	1:8 ^b^ [1:7, 1:11]	1:8 ^b^ [1:7, 1:9]	1:8 ^b^ [1:8, 1:9]	1:5 ^a^ [1:4, 1:5]	1:5 ^a^ [1:5, 1:6]

^1^ Two-way ANOVA analyses were conducted, followed by Tukey HSD’s post hoc test. * *p* < 0.05 significant differences given by the factor “diet” (STD and HFHS); ^$^ *p* < 0.05 significant differences given by the factor “supplement” (CONTROL, FG, ω-3, FG + ω-3). Superscript ^#^ indicates significant interaction (*p* < 0.05) between the factors diet and supplement. Data are means (SD). Ratios are mean [minimum, maximum]. Values with different superscript indicate significant differences (*p* < 0.05) (analyzed by post hoc Tukey HSD).

**Table 4:** FAD indexes and elongases activities from total fatty acid data of kidney and plasma calculated as product/precursor ratio ^1^.

	STD-C	STD-FG	STD-ω3	STD-FG + ω3	HFHS-C	HFHS-FG	HFHS- ω3	HFHS-FG + ω3
Desaturase and elongases activities in kidney								
Elongase-6 (18:0/16:0) *^$#^	0.50 ^b^ (0.14)	0.48 ^b^ (0.06)	0.58 ^ab^ (0.07)	0.70 ^a^ (0.06)	0.69 ^a^ (0.02)	0.67 ^a^ (0.06)	0.64 ^a^ (0.02)	0.68 ^a^ (0.03)
SCD-16 = [16:1ω7/16:0] *^$^	0.11 ^a^ (0.05)	0.10 ^a^ (0.02)	0.08 ^ab^ (0.02)	0.04 ^b^ (0.03)	0.05 ^b^ (0.01)	0.05 ^b^ (0.01)	0.04 ^b^ (0.01)	0.04 ^b^ (0.01)
SCD-18 = [18:1ω9/18:0] *^$#^	1.36 ^a^ (0.92)	1.28 ^ab^ (0.27)	0.74 ^abc^ (0.35)	0.45 ^c^ (0.07)	0.66 ^bc^ (0.08)	0.79 ^abc^ (0.19)	0.71 ^abc^ (0.07)	0.69 ^abc^ (0.11)
Δ4D = [22:6ω3/22:5ω3] *^#^	2.35 ^b^ (0.21)	2.39 ^b^ (0.21)	3.22 ^b^ (0.28)	2.86 ^b^ (0.33)	4.58 ^a^ (0.84)	5.07 ^a^ (0.63)	4.33 ^a^ (0.46)	4.33 ^a^ (0.11)
Δ5D = [20:4ω6/20:3ω6] *^$^	35.24 ^a^ (4.52)	33.09 ^ab^ (3.03)	32.52 ^ab^ (3.21)	32.39 ^ab^ (1.97)	32.65 ^ab^ (1.03)	32.61 ^ab^ (4.67)	27.37 ^b^ (2.22)	26.78 ^b^ (2.99)
Δ6D = [20:3ω6/18:2ω6] *^$#^	0.03 ^cd^ (0.02)	0.02 ^d^ (0.01)	0.04 ^bc^ (0.00)	0.05 ^b^ (0.01)	0.10 ^a^ (0.01)	0.09 ^a^ (0.01)	0.09 ^a^ (0.03)	0.09 ^a^ (0.02)
Δ5D + Δ6D = [20:5ω3/18:3ω3] *^$#^	0.73 ^d^ (0.39)	0.51 ^d^ (0.16)	1.43 ^bc^ (0.33)	1.63 ^bc^ (0.48)	3.17 ^b^ (0.55)	4.25 ^bc^ (0.42)	8.78 ^a^ (1.97)	9.45 ^a^ (2.28)
Desaturase and elongases activities in plasma								
Elongase-6 (18:0/16:0) ^ns^	0.38 (0.03)	0.42 (0.05)	0.39 (0.04)	0.39 (0.11)	0.41 (0.04)	0.44 (0.04)	0.47 (0.15)	0.41 (0.07)
SCD-16 = [16:1ω7/16:0] ^ns^	0.07 (0.01)	0.06 (0.01)	0.07 (0.01)	0.07 (0.01)	0.08 (0.03)	0.07 (0.01)	0.16 (0.21)	0.06 (0.02)
SCD-18 = [18:1ω9/18:0] *	0.86 ^b^ (0.16)	0.73 ^b^ (0.15)	0.84 ^b^ (0.14)	0.88 ^b^ (0.50)	1.60 ^a^ (0.33)	1.46 ^a^ (0.19)	1.61 ^a^ (0.36)	1.47 ^a^ (0.34)
Δ4D = [22:6ω3/22:5ω3] *^$#^	2.93 ^c^ (0.40)	3.10 ^c^ (0.57)	2.63 ^c^ (0.45)	4.13 ^bc^ (0.86)	5.15 ^ab^ (1.18)	4.08 ^bc^ (1.47)	5.81 ^a^ (0.93)	5.57 ^ab^ (0.74)
Δ5D = [20:4ω6/20:3ω6] *^$^	123.48 ^a^ (14.14)	111.62 ^ab^ (10.90)	72.67 ^cd^ (20.84)	83.94 ^bc^ (26.77)	47.94 ^de^ (18.18)	54.77 ^cde^ (12.04)	32.06 ^e^ (6.18)	32.10 ^e^ (5.02)
Δ6D = [20:3ω6/18:2ω6] *^$^	0.01 ^b^ (0.00)	0.01 ^b^ (0.00)	0.02 ^b^ (0.00)	0.02 ^b^ (0.00)	0.05 ^a^ (0.01)	0.05 ^a^ (0.01)	0.06 ^a^ (0.01)	0.06 ^a^ (0.01)
Δ5D + Δ6D = [20:5ω3/18:3ω3] *^$#^	1.85 ^b^ (0.53)	1.97 ^b^ (0.78)	2.95 ^b^ (0.77)	3.09 ^b^ (0.90)	2.30 ^b^ (0.50)	2.27 ^b^ (0.40)	6.14 ^a^ (1.58)	7.32 ^a^ (2.06)

^1^ Two-way ANOVA analyses were conducted, followed by Tukey HSD’s post hoc test. * *p* < 0.05 significant differences given by the factor “diet” (STD and HFHS); ^$^ *p* < 0.05 significant differences given by the factor “supplement” (CONTROL, FG, ω-3, FG + ω-3). Superscript ^#^ indicates significant interaction (*p* < 0.05) between the factors diet and supplement. Means with different superscript indicate significant differences (*p* < 0.05) (analyzed by post hoc Tukey HSD). ns = no significant.

**Table 5:** Carbonylated proteins identified in the kidney of Sprague Dawley rats. Spot Nº refers to numbered spots on Figure S2. Spots of interest were identified by LC-ESI-IT-MS/MS, as described in the Materials and Methods Section.

Spot Nº	Protein ID	Gene Name	Avg. Mass	UniProtKB Code
1	Phosphatidylethanolamine-binding protein 1 OS = Rattus norvegicus OX = 10,116 GN = Pebp1 PE = 1 SV = 3	Pebp1	20,801	P31044|PEBP1_RAT
2	Superoxide dismutase [Mn] mitochondrial OS = Rattus norvegicus OX = 10116 GN = Sod2 PE = 1 SV = 2	Sod2	24,674	P07895|SODM_RAT
3	Glutathione S-transferase alpha-3 OS = Rattus norvegicus OX = 10116 GN = Gsta3 PE = 1 SV = 3	Gsta3	25,319	P04904|GSTA3_RAT
	Glutathione S-transferase P OS = Rattus norvegicus OX = 10116 GN = Gstp1 PE = 1 SV = 2	Gstp1	23,439	P04906|GSTP1_RAT
	Glutathione S-transferase alpha-4 OS = Rattus norvegicus OX = 10116 GN = Gsta4 PE = 1 SV = 2	Gsta4	25,510	P14942|GSTA4_RAT
4	Peroxisomal trans-2-enoyl-CoA reductase OS = Rattus norvegicus OX = 10116 GN = Pecr PE = 2 SV = 1	Pecr	32,433	Q9WVK3|PECR_RAT
5	Triosephosphate isomerase OS = Rattus norvegicus OX = 10116 GN = Tpi1 PE = 1 SV = 2	Tpi1	26,849	P48500|TPIS_RAT
	Electron transfer flavoprotein subunit beta OS = Rattus norvegicus OX = 10116 GN = Etfb PE = 1 SV = 3	Etfb	27,687	Q68FU3|ETFB_RAT
6	Enoyl-CoA delta isomerase 1 mitochondrial OS = Rattus norvegicus OX = 10116 GN = Eci1 PE = 1 SV = 1	Eci1	32,254	P23965|ECI1_RAT
7	Carbonic anhydrase 2 OS = Rattus norvegicus OX = 10116 GN = Ca2 PE = 1 SV = 2	Ca2	29,114	P27139|CAH2_RAT
8	Omega-amidase NIT2 OS = Rattus norvegicus OX = 10116 GN = Nit2 PE = 1 SV = 1	Nit2	30,701	Q497B0|NIT2_RAT
9	3-hydroxyisobutyrate dehydrogenase mitochondrial OS = Rattus norvegicus OX = 10116 GN = Hibadh PE = 1 SV = 3	Hibadh	35,303	P29266|3HIDH_RAT
10	Malate dehydrogenase cytoplasmic OS = Rattus norvegicus OX = 10116 GN = Mdh1 PE = 1 SV = 3	Mdh1	36,483	O88989|MDHC_RAT
11	Actin cytoplasmic 1 OS = Rattus norvegicus OX = 10116 GN = Actb PE = 1 SV = 1	Actb	41,737	P60711|ACTB_RAT
12	Heat shock cognate 71 kDa protein OS = Rattus norvegicus OX = 10116 GN = Hspa8 PE = 1 SV = 1	Hspa8	70,871	P63018|HSP7C_RAT
13	Fructose-bisphosphate aldolase B OS = Rattus norvegicus OX = 10116 GN = Aldob PE = 1 SV = 2	Aldob	39,618	P00884|ALDOB_RAT
14	Aspartate aminotransferase mitochondrial OS = Rattus norvegicus OX = 10116 GN = Got2 PE = 1 SV = 2	Got2	47,314	P00507|AATM_RAT
15	Malate dehydrogenase mitochondrial OS = Rattus norvegicus OX = 10116 GN = Mdh2 PE = 1 SV = 2	Mdh2	35,684	P04636|MDHM_RAT
16	Glyceraldehyde-3-phosphate dehydrogenase OS = Rattus norvegicus OX = 10116 GN = Gapdh PE = 1 SV = 3	Gapdh	35,828	P04797|G3P_RAT
17	Hydroxyacyl-coenzyme A dehydrogenase mitochondrial OS = Rattus norvegicus OX = 10116 GN = Hadh PE = 2 SV = 1	Hadh	34,448	Q9WVK7|HCDH_RAT
18	Hydroxyacid oxidase 2 OS = Rattus norvegicus OX = 10116 GN = Hao2 PE = 1 SV = 2	Hao2	39,201	Q07523|HAOX2_RAT
19	Aflatoxin B1 aldehyde reductase member 2 OS = Rattus norvegicus OX = 10116 GN = Akr7a2 PE = 1 SV = 2	Akr7a2	40,675	Q8CG45|ARK72_RAT
20	Aldo-keto reductase family 1 member A1 OS = Rattus norvegicus OX = 10116 GN = Akr1a1 PE = 1 SV = 2	Akr1a1	36,506	P51635|AK1A1_RAT
21	Sorbitol dehydrogenase OS = Rattus norvegicus OX = 10116 GN = Sord PE = 1 SV = 4	Sord	38,235	P27867|DHSO_RAT
22	Aspartate aminotransferase cytoplasmic OS = Rattus norvegicus OX = 10116 GN = Got1 PE = 1 SV = 3	Got1	46,429	P13221|AATC_RAT
23	Isocitrate dehydrogenase [NADP] cytoplasmic OS = Rattus norvegicus OX = 10116 GN = Idh1 PE = 1 SV = 1	Idh1	46,734	P41562|IDHC_RAT
24	Aminoacylase-1A OS = Rattus norvegicus OX = 10116 GN = Acy1a PE = 1 SV = 1	Acy1a	45,804	Q6AYS7|ACY1A_RAT
25	Aldehyde dehydrogenase mitochondrial OS = Rattus norvegicus OX = 10116 GN = Aldh2 PE = 1 SV = 1	Aldh2	56,488	P11884|ALDH2_RAT
	4-trimethylaminobutyraldehyde dehydrogenase OS = Rattus norvegicus OX = 10116 GN = Aldh9a1 PE = 1 SV = 1	Aldh9a1	53,653	Q9JLJ3|AL9A1_RAT
26	Serum albumin OS = Rattus norvegicus OX = 10116 GN = Alb PE = 1 SV = 2	Alb	68,731	P02770|ALBU_RAT
27	Protein disulfide-isomerase A3 OS = Rattus norvegicus OX = 10116 GN = Pdia3 PE = 1 SV = 2	Pdia3	56,623	P11598|PDIA3_RAT
	Alpha-aminoadipic semialdehyde dehydrogenase OS = Rattus norvegicus OX = 10116 GN = Aldh7a1 PE = 1 SV = 2	Aldh7a1	58,749	Q64057|AL7A1_RAT
28	Glutamate dehydrogenase 1 mitochondrial OS = Rattus norvegicus OX = 10116 GN = Glud1 PE = 1 SV = 2	Glud1	61,416	P10860|DHE3_RAT
29	Alanine--glyoxylate aminotransferase 2 mitochondrial OS = Rattus norvegicus OX = 10116 GN = Agxt2 PE = 1 SV = 2	Agxt2	57,201	Q64565|AG_RAT
30	Retinal dehydrogenase 1 OS = Rattus norvegicus OX = 10116 GN = Aldh1a1 PE = 1 SV = 3	Aldh1a1	54,459	P51647|AL1A1_RAT
31	Succinyl-CoA:3-ketoacid coenzyme A transferase 1 mitochondrial OS = Rattus norvegicus OX = 10116 GN = Oxct1 PE = 1 SV = 1	Oxct1	56,204	B2GV06|SCOT1_RAT
	Dihydrolipoyl dehydrogenase mitochondrial OS = Rattus norvegicus OX = 10116 GN = Dld PE = 1 SV = 1	Dld	54,038	Q6P6R2|DLDH_RAT
	Methylmalonate-semialdehyde dehydrogenase [acylating] mitochondrial OS = Rattus norvegicus OX = 10116 GN = Aldh6a1 PE = 1 SV = 1	Aldh6a1	57,808	Q02253|MMSA_RAT
32	Catalase OS = Rattus norvegicus OX = 10116 GN = Cat PE = 1 SV = 3	Cat	59,757	P04762|CATA_RAT
	Triokinase/FMN cyclase OS = Rattus norvegicus OX = 10116 GN = Tkfc PE = 1 SV = 1	Tkfc	59,444	Q4KLZ6|TKFC_RAT
33	Acyl-coenzyme A synthetase ACSM2 mitochondrial OS = Rattus norvegicus OX = 10116 GN = Acsm2 PE = 2 SV = 2	Acsm2	64,145	O70490|ACSM2_RAT
	Transketolase OS = Rattus norvegicus OX = 10116 GN = Tkt PE = 1 SV = 1	Tkt	67,644	P50137|TKT_RAT
34	Aconitate hydratase mitochondrial OS = Rattus norvegicus OX = 10116 GN = Aco2 PE = 1 SV = 2	Aco2	85,433	Q9ER34|ACON_RAT
35	Serotransferrin OS = Rattus norvegicus OX = 10116 GN = Tf PE = 1 SV = 3	Tf	76,395	P12346|TRFE_RAT
36	Cytoplasmic aconitate hydratase OS = Rattus norvegicus OX = 10116 GN = Aco1 PE = 1 SV = 1	Aco1	98,127	Q63270|ACOC_RAT

**Table 6:** KEGG pathway enrichment analysis for renal proteins was significantly more carbonylated due to the HFHS-diet intake compared to STD-diet (red), and for renal proteins, the analysis was significantly less carbonylated due to the supplementation of the HFHS diet with FG, fish oil, and both (green) ^1^.

ID	KEGG Pathway	Effect of HFHS	Effect of FG	Effect of ω3	Effect of FG + ω3
rno04964	Proximal tubule bicarbonate reclamation	6.10 × 10^−3^			
rno00910	Nitrogen metabolism	4.60 × 10^−3^			
rno01230	Biosynthesis of amino acids	3.61 × 10^−8^	0.00051	5.05 × 10^−8^	1.28 × 10^−6^
rno00400	Phenylalanine, tyrosine and tryptophan biosynthesis				
rno00360	Phenylalanine metabolism				
rno00350	Tyrosine metabolism				
rno00380	Tryptophan metabolism	3.20 × 10^−7^	0.0116		0.00083
rno00280	Valine, leucine and isoleucine degradation	8.40 × 10^−9^	0.00028		0.0222
rno00250	Alanine, aspartate and glutamate metabolism	0.0114		0.0062	0.0117
rno00410	beta-Alanine metabolism	1.90 × 10^−4^			
rno00260	Glycine, serine and threonine metabolism				
rno00330	Arginine and proline metabolism	0.00077			0.0222
rno00220	Arginine biosynthesis	8.47 × 10^−5^		0.003	0.0056
rno00270	Cysteine and methionine metabolism				
rno00340	Histidine metabolism	0.0072			
rno00310	Lysine degradation	4.07 × 10^−5^			0.0258
rno01200	Carbon metabolism	2.86 × 10^−16^	1.59 × 10^−13^	1.59 × 10^−13^	5.01× 10^−13^
rno00010	Glycolysis/Gluconeogenesis	1.44 × 10^−8^	0.00034	0.00034	3.51 × 10^−5^
rno00020	Citrate cycle (TCA cycle)	9.20 × 10^−3^	0.0066		
rno00620	Pyruvate metabolism	3.10 × 10^−4^			
rno01210	2-Oxocarboxylic acid metabolism	6.91 × 10^−5^		0.0028	0.0055
rno00030	Pentose phosphate pathway	0.0092		0.0054	0.0099
rno00040	Pentose and glucuronate interconversions	9.20 × 10^−3^		0.0054	0.0099
rno00051	Fructose and mannose metabolism	0.00026	0.0001	7.81 × 10^−5^	0.00038
rno00630	Glyoxylate and dicarboxylate metabolism	2.10 × 10^−4^	0.0066		0.0099
rno00071	Fatty acid degradation	2.04 × 10^−5^			0.00083
rno00561	Glycerolipid metabolism	5.00 × 10^−5^		0.0174	1.50 × 10^−3^
rno00650	Butanoate metabolism	0.00016			0.0086
rno00640	Propanoate metabolism	0.0099	0.0066		
rno00053	Ascorbate and aldarate metabolism	0.0061			
rno00480	Glutathione metabolism				
rno00980	Metabolism of xenobiotics by cytochrome P450				
rno04146	Peroxisome	0.0028	0.0287	0.0263	2.80 × 10^−3^
rno04213	Longevity regulating pathway—multiple species	0.0323			0.0281
rno05418	Fluid shear stress and atherosclerosis				
rno04066	HIF-1 signaling pathway	5.90 × 10^−3^	0.0465	0.0429	0.0055

^1^ KEGG pathway enrichment was conducted by using STRING. Only pathways significantly enriched at FDR< 5% are considered. FDR values of each pathway are shown in table. FDR: False Discovery Rate.

## Data Availability

The data presented in this study are available in the article and Supplementary Materials.

## Supplementary Materials

The following supporting information can be downloaded at: https://www.mdpi.com/article/10.3390/antiox12030751/s1, Figure S1. Total proteins carbonylation in rat kidneys. Upper panels show representative images of FTSC-label carbonylated proteins resolved in 1D gels from (A) STD-CONTROL, (B) STD-FG, (C) STD-ω3, (D) STD-FG + ω3, (E) HFHS-CONTROL, (F) HFHS-FG, (G) HFHS-ω3, and (H) HFHS-FG + ω3. Lower panels show their corresponding Coomassie-stained 1D gels. Images are representatives of three independent labelling experiments performed in triplicates. STD (standard diet), HFHS (high-fat high-sucrose diet), ω3 (supplementation with fish oil), and FG (supplementation with D-Fagomine), Figure S2. Carbonylated proteins identified in rat kidneys. Upper panels show representative images of FTSC label carbonylated proteins resolved in 2D gels from (A) STD-CONTROL, (B) STD-FG, (C) STD-ω3, (D) STD-FG + ω3, (E) HFHS-CONTROL, (F) HFHS-FG, (G) HFHS-ω3, (H) HFHS-FG + ω3. Lower panels show their corresponding Coomassie-stained 2D gels. Numbered protein spots represent carbonylated proteins confidently identified, and they are listed in Table 5 and Supplementary Table S3. Images are representatives of three independent labelling experiments performed in triplicates. STD (standard diet), HFHS (high-fat high-sucrose diet), ω3 (supplementation with fish oil); FG (supplementation with D-fagomine); Table S1: Diet composition; Table S2: Fatty acid composition of each diet (chow + supplement). Results are expressed as a percentage of total fatty acids (mg/100 mg of Total FA); Table S3: Carbonylated proteins identified by nanoLC-MS/MS in the kidney. Carbonylation index calculated for each protein in each diet are also showed. Protein Spot No. refers to the numbered spots in 2D gels shown in Figure S2.
