# Supplementary material for: Combined Intake of Fish Oil and D-Fagomine Prevents High-Fat High-Sucrose Diet-Induced Prediabetes by Modulating Lipotoxicity and Protein Carbonylation in the Kidney
Source: Antioxidants (Basel). 2023 Mar 19;12(3):751. doi: 10.3390/antiox12030751 (PMC10045798; doi:10.3390/antiox12030751)
Supplement: Supplementary file 1 [file antioxidants-12-00751-s001.zip › antioxidants-2274999-supplementary.pdf]

Supplementary Materials

Table S1. Diet composition.

	**STD[a]-C/-FG**	**STD[a]-ω3/-FG+ω3**	**[b]HFHS-C/-FG**	**[b]HFHS-ω3/-FG+ω3**
Flour (g)	1000[a]	1000[a]	1000[b]	1000[b]
Porcine gelatin (g)	25	25	25	25
Soybean lecithin (g)	6	6	22	22
Oil (mL)	19 mL soybean	19 mL EPA:DHA 1:1	24 mL soybean	24 mL EPA:DHA 1:1
Protein (% weight)	16.4	16.4	21.7	21.7
Fat (% weight)	6.2	6.2	24.1	24.1
Carbohydrates (% weight)	46.6	46.6	45.0	45.0
Total energy density (kcal/g)	3.1	3.1	4.8	4.8

[a]Teklad Global 14% Protein Rodent Maintenance Diet (Envigo, IN, USA). [b]TD.08811 45% Kcal Fat Diet (Envigo, IN, USA). STD (standard diet), HFHS (high-fat high-sucrose diet), ω3 (supplementation with fish oil); FG (supplementation with D-Fagomine).

Table S2. Fatty acid composition of each diet (chow + supplement). Results are expressed as a percentage of total fatty acids (mg/100mg of Total FA).

**FATTY ACIDS**	**STD-C/-FG**	**STD-ω3/-FG+ω3**	**HFHS-C/-FG**	**HFHS-ω3/-FG+ω3**
14:00	0.0	0.1	11.5	11.6
16:00	14.8	14.6	32.2	32.0
16:1ω7	0.0	0.1	1.7	1.7
18:00	2.9	2.9	13.7	13.7
18:1ω9	20.5	20.3	26.4	26.2
18:1ω7	0.0	0.0	0.7	0.7
18:2ω6	58.6	57.8	9.7	8.7
20:00	n.d.	0.0	n.d.	0.0
18:3ω3	2.9	2.9	1.1	1.1
20:1ω9	0.0	0.0	0.0	0.0
18:4ω3	0.0	0.0	0.0	0.0
20:2ω6	0.0	0.0	0.0	0.0
20:3ω6	n.d.	0.0	n.d.	0.0
20:4ω6	0.0	0.0	0.0	0.0
22:1ω11	0.0	0.0	0.0	0.0
22:1ω9	0.0	0.0	0.0	0.0
20:4ω3	0.0	0.0	0.0	0.0
20:5ω3	0.0	0.4	0.0	0.5
24:1ω9	0.0	0.0	0.0	0.0
22:5ω3	0.0	0.1	0.0	0.1
22:6ω3	0.0	0.4	0.0	0.6
**ω3**	3.3	3.2	1.6	1.4
**ω6**	58.8	58.9	10.4	10.6
**ω6/ω3**	17.8	18.4	6.5	7.6
**SFAs**	17.5	18.3	59.4	60.5
**MUFAs**	21.1	20.3	29.3	28.4
**PUFAs**	61.1	61.0	10.8	10.7
**EPA/DHA**	-	1/1	-	1/1.2

[1] STD (standard diet), HFHS (high-fat high-sucrose diet), ω3 (supplementation with fish oil); FG (supplementation with D-Fagomine); SFAs (saturated fatty acids); MUFAs (monounsaturated fatty acids); PUFAs (polyunsaturated fatty acids); EPA (eicosapentaenoic fatty acid); DHA (docosahexaenoic fatty acid).

Table S3. Carbonylated proteins identified by nanoLC-MS/MS in kidney. Carbonylation index calculated for each protein in each diet are also showed. Protein Spot No. refers to the numbered spots in 2D gels shown in Figure S2.

Spot Nº	Protein ID	Gene Name	Avg. Mass	UniProtKB code	STD-C	STD-FG	STD-ω3	STD-FG+ω3	HFHS-C	HFHS-FG	HFHS-ω3	HFHS-FG+ω3
1	Phosphatidylethanolamine-binding protein 1 OS=Rattus norvegicus OX=10116 GN=Pebp1 PE=1 SV=3	Pebp1	20801	P31044∣PEBP1_RAT	1.02^{c}(0.01)	1.14^{c}(0.14)	0.97^{c}(0.17)	1.07^{c}(0.25)	1.51^{ab}(0.01)	1.58^{a}(0.13)	1.11^{c}(0.02)	1.17^{bc}(0.06)
2	Superoxide dismutase [Mn] mitochondrial OS=Rattus norvegicus OX=10116 GN=Sod2 PE=1 SV=2	Sod2	24674	P07895∣SODM_RAT	1.88(0.50)	1.86(0.22)	1.94(0.58)	1.85(0.17)	2.03(0.44)	1.97(0.15)	2.12(0.22)	2.03(0.12)
3	Glutathione S-transferase alpha-3 OS=Rattus norvegicus OX=10116 GN=Gsta3 PE=1 SV=3	Gsta3	25319	P04904∣GSTA3_RAT	0.36(0.10)	0.38(0.21)	0.37(0.17)	0.34(0.23)	0.38(0.09)	0.35(0.13)	0.38(0.10)	0.37(0.12)
	Glutathione S-transferase P OS=Rattus norvegicus OX=10116 GN=Gstp1 PE=1 SV=2	Gstp1	23439	P04906∣GSTP1_RAT								
	Glutathione S-transferase alpha-4 OS=Rattus norvegicus OX=10116 GN=Gsta4 PE=1 SV=2	Gsta4	25510	P14942∣GSTA4_RAT								
4	Peroxisomal trans-2-enoyl-CoA reductase OS=Rattus norvegicus OX=10116 GN=Pecr PE=2 SV=1	Pecr	32433	Q9WVK3∣PECR_RAT	0.71(0.22)	0.73(0.25)	0.73(0.31)	0.72(0.34)	0.71(0.18)	0.59(0.15)	0.72(0.15)	0.72(0.20)
5	Triosephosphate isomerase OS=Rattus norvegicus OX=10116 GN=Tpi1 PE=1 SV=2	Tpi1	26849	P48500∣TPIS_RAT	0.54^{abc}(0.13)	0.29^{c}(0.07)	0.37^{abc}(0.06)	0.29^{c}(0.08)	0.62^{a}(0.16)	0.35^{bc}(0.00)	0.61^{ab}(0.02)	0.33^{c}(0.11)
	Electron transfer flavoprotein subunit beta OS=Rattus norvegicus OX=10116 GN=Etfb PE=1 SV=3	Etfb	27687	Q68FU3∣ETFB_RAT								
6	Enoyl-CoA delta isomerase 1 mitochondrial OS=Rattus norvegicus OX=10116 GN=Eci1 PE=1 SV=1	Eci1	32254	P23965∣ECI1_RAT	0.50^{ab}(0.01)	0.36^{bc}(0.02)	0.47^{abc}(0.05)	0.28^{c}(0.08)	0.57^{a}(0.08)	0.36^{bc}(0.03)	0.56^{a}(0.13)	0.44^{abc}(0.07)
7	Carbonic anhydrase 2 OS=Rattus norvegicus OX=10116 GN=Ca2 PE=1 SV=2	Ca2	29114	P27139∣CAH2_RAT	0.95^{b}(0.06)	0.87^{b}(0.06)	0.90^{b}(0.06)	0.92^{b}(0.18)	1.87^{a}(0.29)	1.70^{a}(0.29)	1.50^{a}(0.19)	1.49^{a}(0.14)
8	Omega-amidase NIT2 OS=Rattus norvegicus OX=10116 GN=Nit2 PE=1 SV=1	Nit2	30701	Q497B0∣NIT2_RAT	0.62(0.24)	0.57(0.31)	0.61(0.22)	0.56(0.42)	0.66(0.35)	0.55(0.29)	0.67(0.16)	0.66(0.04)
9	3-hydroxyisobutyrate dehydrogenase mitochondrial OS=Rattus norvegicus OX=10116 GN=Hibadh PE=1 SV=3	Hibadh	35303	P29266∣3HIDH_RAT	0.62(0.24)	0.60(0.34)	0.61(0.28)	0.60(0.34)	0.60(0.30)	0.53(0.25)	0.63(0.17)	0.62(0.10)
10	Malate dehydrogenase cytoplasmic OS=Rattus norvegicus OX=10116 GN=Mdh1 PE=1 SV=3	Mdh1	36483	O88989∣MDHC_RAT	0.27(0.08)	0.31(0.19)	0.43(0.03)	0.32(0.37)	0.27(0.06)	0.23(0.05)	0.28(0.12)	0.12(0.04)
11	Actin cytoplasmic 1 OS=Rattus norvegicus OX=10116 GN=Actb PE=1 SV=1	Actb	41737	P60711∣ACTB_RAT	0.19^{b}(0.04)	0.22^{ab}(0.09)	0.21^{ab}(0.14)	0.20^{b}(0.08)	0.58^{a}(0.15)	0.56^{ab}(0.20)	0.37^{ab}(0.22)	0.26^{ab}(0.04)
12	Heat shock cognate 71 kDa protein OS=Rattus	Hspa8	70871	P63018∣HSP7C_RAT	0.64^{ab}(0.26)	0.66^{ab}(0.29)	0.63^{ab}(0.17)	0.56^{b}(0.20)	1.39^{a}(0.37)	1.16^{ab}(0.32)	0.90^{ab}(0.35)	0.86^{ab}(0.057)

	norvegicus OX=10116 GN=Hspa8 PE=1 SV=1											
13	Fructose-bisphosphate aldolase B OS=Rattus norvegicus OX=10116 GN=Aldob PE=1 SV=2	Aldob	39618	P00884∣ALDOB_RAT	0.31^{cd}(0.03)	0.23^{d}(0.05)	0.25^{d}(0.06)	0.24^{d}(0.07)	0.85^{a}(0.13)	0.61^{b}(0.01)	0.50^{b}(0.02)	0.45^{bc}(0.02)
14	Aspartate aminotransferase mitochondrial OS=Rattus norvegicus OX=10116 GN=Got2 PE=1 SV=2	Got2	47314	P00507∣AATM_RAT	0.79(0.08)	0.75(0.33)	0.75(0.35)	0.71(0.41)	1.17(0.21)	0.97(0.18)	0.83(0.19)	0.83(0.09)
15	Malate dehydrogenase mitochondrial OS=Rattus norvegicus OX=10116 GN=Mdh2 PE=1 SV=2	Mdh2	35684	P04636∣MDHM_RAT	0.28(0.16)	0.28(0.23)	0.28(0.11)	0.27(0.11)	0.34(0.06)	0.28(0.05)	0.39(0.18)	0.39(0.10)
16	Glyceraldehyde-3-phosphate dehydrogenase OS=Rattus norvegicus OX=10116 GN=Gapdh PE=1 SV=3	Gapdh	35828	P04797∣G3P_RAT	0.36^{bc}(0.04)	0.18^{d}(0.10)	0.25^{cd}(0.03)	0.17^{d}(0.04)	0.79^{a}(0.01)	0.67^{a}(0.01)	0.46^{b}(0.08)	0.48^{b}(0.07)
17	Hydroxyacyl-coenzyme A dehydrogenase mitochondrial OS=Rattus norvegicus OX=10116 GN=Hadh PE=2 SV=1	Hadh	34448	Q9WVK7∣HCDH_RAT	0.46^{abc}(0.17)	0.25^{c}(0.03)	0.32^{bc}(0.07)	0.32^{bc}(0.069)	0.79^{a}(0.09)	0.65^{ab}(0.08)	0.56^{abc}(0.22)	0.68^{a}(0.16)
18	Hydroxyacid oxidase 2 OS=Rattus norvegicus OX=10116 GN=Hao2 PE=1 SV=2	Hao2	39201	Q07523∣HAOX2_RAT	0.38^{b}(0.07)	0.36^{b}(0.15)	0.34^{b}(0.10)	0.34^{b}(0.087)	0.72^{a}(0.09)	0.72^{a}(0.09)	0.49^{ab}(0.12)	0.46^{ab}(0.10)
19	Aflatoxin B1 aldehyde reductase member 2 OS=Rattus norvegicus OX=10116 GN=Akr7a2 PE=1 SV=2	Akr7a2	40675	Q8CG45∣ARK72_RAT	0.64(0.17)	0.61(0.14)	0.65(0.08)	0.57(0.28)	0.69(0.14)	0.65(0.22)	0.52(0.01)	0.54(0.07)
20	Aldo-keto reductase family 1 member A1 OS=Rattus norvegicus OX=10116 GN=Akr1a1 PE=1 SV=2	Akr1a1	36506	P51635∣AK1A1_RAT	0.45^{b}(0.14)	0.42^{b}(0.22)	0.21^{b}(0.10)	0.39^{b}(0.14)	1.35^{a}(0.16)	1.30^{a}(0.21)	0.49^{b}(0.15)	0.45^{b}(0.18)
21	Sorbitol dehydrogenase OS=Rattus norvegicus OX=10116 GN=Sord PE=1 SV=4	Sord	38235	P27867∣DHSO_RAT	0.51^{bcd}(0.09)	0.33^{cd}(0.11)	0.30^{d}(0.01)	0.29^{d}(0.01)	0.92^{a}(0.10)	0.70^{ab}(0.05)	0.57^{bc}(0.14)	0.55^{bc}(0.09)
22	Aspartate aminotransferase cytoplasmic OS=Rattus norvegicus OX=10116 GN=Got1 PE=1 SV=3	Got1	46429	P13221∣AATC_RAT	0.45^{c}(0.10)	0.44^{c}(0.14)	0.70^{bc}(0.01)	0.39^{c}(0.18)	1.09^{a}(0.22)	0.10^{ab}(0.12)	0.44^{c}(0.05)	0.43^{c}(0.07)
23	Isocitrate dehydrogenase [NADP] cytoplasmic OS=Rattus norvegicus OX=10116 GN=Idh1 PE=1 SV=1	Idh1	46734	P41562∣IDHC_RAT	0.26^{c}(0.09)	0.24^{c}(0.04)	0.55^{b}(0.03)	0.26^{c}(0.06)	0.98^{a}(0.25)	0.46^{bc}(0.00)	0.68^{b}(0.08)	0.56^{b}(0.05)
24	Aminoacylase-1A OS=Rattus norvegicus OX=10116 GN=Acy1a PE=1 SV=1	Acy1a	45804	Q6AYS7∣ACY1A_RAT	0.48^{a}(0.22)	0.49^{a}(0.29)	0.45^{a}(0.29)	0.46^{a}(0.35)	0.85^{a}(0.22)	0.86^{a}(0.04)	0.65^{a}(0.29)	0.64^{a}(0.10)
25	Aldehyde dehydrogenase mitochondrial OS=Rattus norvegicus OX=10116 GN=Aldh2 PE=1 SV=1	Aldh2	56488	P11884∣ALDH2_RAT	0.48^{a}(0.19)	0.42^{a}(0.21)	0.45^{a}(0.29)	0.42^{a}(0.09)	0.71^{a}(0.05)	0.58^{a}(0.03)	0.70^{a}(0.05)	0.53^{a}(0.07)
	4-trimethylaminobutyraldehyde dehydrogenase OS=Rattus norvegicus OX=10116 GN=Aldh9a1 PE=1 SV=1	Aldh9a1	53653	Q9JLJ3∣AL9A1_RAT								
26	Serum albumin OS=Rattus norvegicus OX=10116 GN=Alb PE=1 SV=2	Alb	68731	P02770∣ALBU_RAT	0.47^{cd}(0.05)	0.39^{d}(0.21)	0.66^{abc}(0.06)	0.60^{bcd}(0.02)	0.87^{a}(0.02)	0.74^{ab}(0.02)	0.42^{d}(0.02)	0.42^{d}(0.07)
27	Protein disulfide-isomerase A3 OS=Rattus norvegicus OX=10116 GN=Pdia3 PE=1 SV=2	Pdia3	56623	P11598∣PDIA3_RAT	0.40^{abc}(0.08)	0.73^{a}(0.07)	0.38^{bc}(0.16)	0.36^{c}(0.13)	0.43^{abc}(0.16)	0.36^{bc}(0.12)	0.41^{abc}(0.17)	0.71^{ab}(0.03)
	Alpha-aminoadipic semialdehyde dehydrogenase OS=Rattus norvegicus OX=10116 GN=Aldh7a1 PE=1	Aldh7a1	58749	Q64057∣AL7A1_RAT								

	SV=2										
28	Glutamate dehydrogenase 1 mitochondrial OS=Rattus norvegicus OX=10116 GN=Glud1 PE=1 SV=2	Glud1	61416	P10860 l DHE3_RAT	0.43^{abc}(0.03)	0.27^{cd}(0.09)	0.33^{bcd}(0.12)	0.23^{d}(0.12)	0.62^{a}(0.04)	0.49^{ab}(0.01)	0.46^{abc}(0.04)	0.41^{bcd}(0.01)
29	Alanine--glyoxylate aminotransferase 2 mitochondrial OS=Rattus norvegicus OX=10116 GN=Agxt2 PE=1 SV=2	Agxt2	57201	Q64565 l AG_RAT	0.38(0.08)	0.37(0.07)	0.41(0.19)	0.40(0.17)	0.40(0.08)	0.38(0.02)	0.41(0.11)	0.40(0.02)
30	Retinal dehydrogenase 1 OS=Rattus norvegicus OX=10116 GN=Aldh1a1 PE=1 SV=3	Aldh1a1	54459	P51647 l AL1A1_RAT	0.75^{a}(0.09)	0.55^{a}(0.39)	0.62^{a}(0.14)	0.63^{a}(0.36)	1.17^{a}(0.21)	1.06^{a}(0.03)	0.81^{a}(0.08)	0.78^{a}(0.22)
31	Succinyl-CoA:3-ketoacid coenzyme A transferase 1 mitochondrial OS=Rattus norvegicus OX=10116 GN=Oxct1 PE=1 SV=1	Oxct1	56204	B2GV06 l SCOT1_RAT	0.60^{a}(0.12)	0.38^{a}(0.06)	0.41^{a}(0.06)	0.34^{a}(0.04)	0.71^{a}(0.25)	0.40^{a}(0.04)	0.69^{a}(0.20)	0.69^{a}(0.15)
	Dihydrolipoyl dehydrogenase mitochondrial OS=Rattus norvegicus OX=10116 GN=Dld PE=1 SV=1	Dld	54038	Q6P6R2 l DLDH_RAT								
	Methylmalonate-semialdehyde dehydrogenase [acylating] mitochondrial OS=Rattus norvegicus OX=10116 GN=Aldh6a1 PE=1 SV=1	Aldh6a1	57808	Q02253 l MMSA_RAT								
32	Catalase OS=Rattus norvegicus OX=10116 GN=Cat PE=1 SV=3	Cat	59757	P04762 l CATA_RAT	0.46^{b}(0.16)	0.31^{b}(0.16)	0.26^{b}(0.09)	0.27^{b}(0.08)	0.89^{a}(0.16)	0.60^{ab}(0.09)	0.51^{b}(0.11)	0.51^{b}(0.10)
	Triokinase/FMN cyclase OS=Rattus norvegicus OX=10116 GN=Tkfc PE=1 SV=1	Tkfc	59444	Q4KLZ6 l TKFC_RAT								
33	Acyl-coenzyme A synthetase ACSM2 mitochondrial OS=Rattus norvegicus OX=10116 GN=Acsm2 PE=2 SV=2	Acsm2	64145	O70490 l ACSM2_RAT	0.71^{b}(0.25)	0.66^{b}(0.26)	0.56^{b}(0.49)	0.65^{b}(0.40)	2.06^{a}(0.21)	1.40^{ab}(0.25)	1.04^{b}(0.17)	0.75^{b}(0.24)
	Transketolase OS=Rattus norvegicus OX=10116 GN=Tkt PE=1 SV=1	Tkt	67644	P50137 l TKT_RAT								
34	Aconitate hydratase mitochondrial OS=Rattus norvegicus OX=10116 GN=Aco2 PE=1 SV=2	Aco2	85433	Q9ER34 l ACON_RAT	0.49(0.00)	0.50(0.24)	0.51(0.16)	0.53(0.41)	0.54(0.13)	0.63(0.08)	0.53(0.27)	0.54(0.07)
35	Serotransferrin OS=Rattus norvegicus OX=10116 GN=Tf PE=1 SV=3	Tf	76395	P12346 l TRFE_RAT	0.22^{ab}(0.14)	0.19^{ab}(0.04)	0.21^{ab}(0.10)	0.19^{b}(0.04)	0.35^{ab}(0.08)	0.45^{a}(0.11)	0.35^{ab}(0.09)	0.34^{ab}(0.08)
36	Cytoplasmic aconitate hydratase OS=Rattus norvegicus OX=10116 GN=Aco1 PE=1 SV=1	Aco1	98127	Q63270 l ACOC_RAT	0.14(0.01)	0.16(0.08)	0.18(0.14)	0.16(0.12)	0.16(0.03)	0.30(0.07)	0.15(0.06)	0.16(0.17)

[1] Two-way ANOVA analyses were conducted, followed by Tukey HSD´s post hoc test. * $p<0.05$ significant differences given by the factor "diet" (STD and HFHS); $ $p<0.05$ significant differences given by the factor "supplement" (CONTROL, FG, ω-3, FG+ ω-3). Superscript # indicates significant interaction ($p<0.05$) between the factors diet and supplement. Means with different superscript indicate significant differences ($p<0.05$) (analyzed by post hoc Tukey HSD).

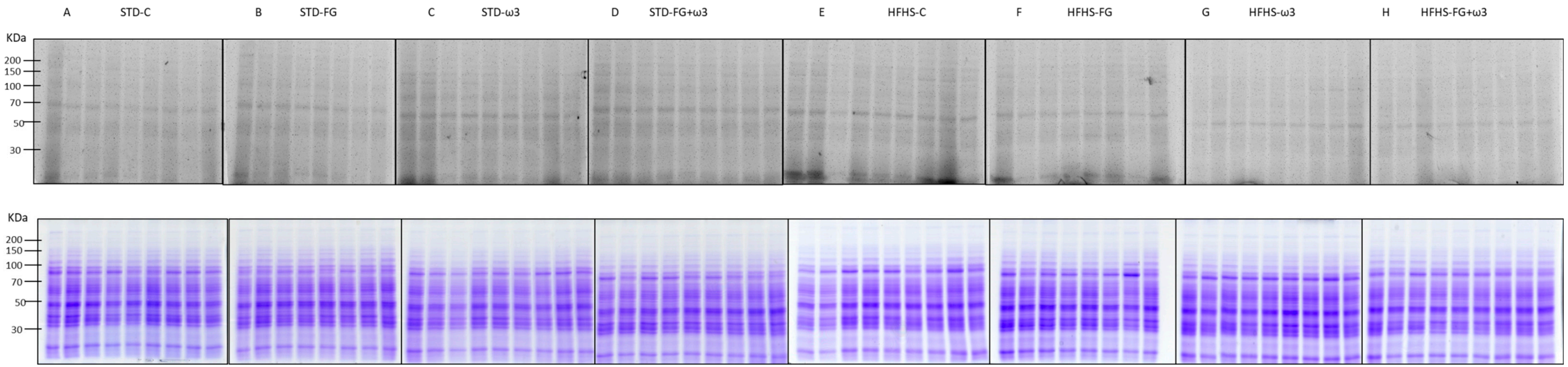

Figure S1. Total proteins carbonylation in rat kidneys. Upper panels show representative images of FTSC-label carbonylated proteins resolved in 1D gels from (**A**) STD-CONTROL, (**B**) STD-FG, (**C**) STD-ω3, (**D**) STD-FG+ω3, (**E**) HFHS-CONTROL, (**F**) HFHS-FG, (**G**) HFHS-ω3, (**H**) HFHS-FG+ω3. Lower panels show their corresponding Coomassie stained 1D gels. Images are representatives of three independent labelling experiments performed in triplicates. STD (standard diet), HFHS (high-fat high-sucrose diet), ω3 (supplementation with fish oil); FG (supplementation with D-Fagomine).

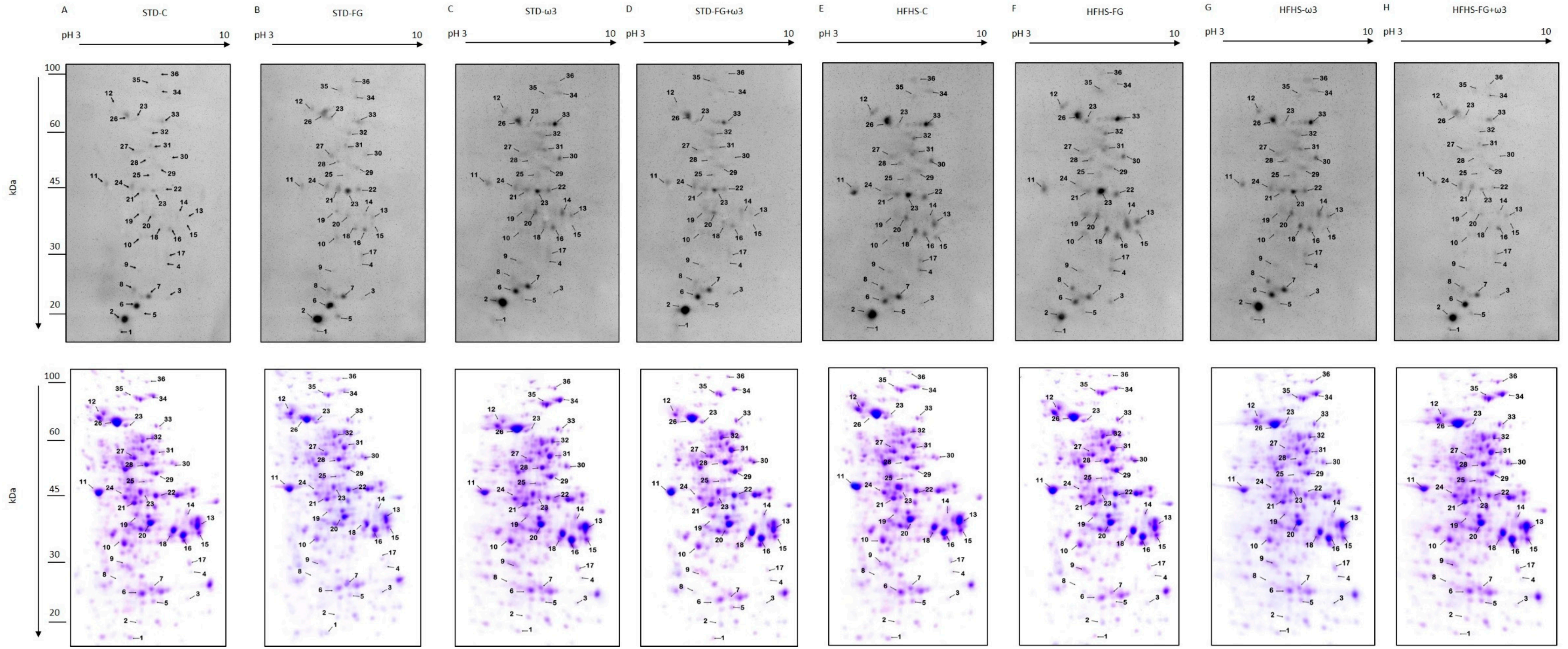

Figure S2. Carbonylated proteins identified in rat kidneys. Upper panels show representative images of FTSC-label carbonylated proteins resolved in 2D gels from (**A**) STD-CONTROL, (**B**) STD-FG, (**C**) STD-ω3, (**D**) STD-FG+ω3, (**E**) HFHS-CONTROL, (**F**) HFHS-FG, (**G**) HFHS-ω3, (**H**) HFHS-FG+ω3. Lower panels show their corresponding Coomassie stained 2D gels. Numbered protein spots represent carbonylated proteins confidently identified, and they are listed in Table 5 and Supplementary Table S3. Images are representatives of three independent labelling experiments performed in triplicates. STD (standard diet), HFHS (high-fat high-sucrose diet), ω3 (supplementation with fish oil); FG (supplementation with D-Fagomine).
